# Threshold of anthropogenic sound levels within protected landscapes in Kerala, India, for avian habitat quality and conservation

**DOI:** 10.1038/s41598-024-53153-6

**Published:** 2024-02-01

**Authors:** Sajeev C. Rajan, Vishnu M, Ahalya Mitra, Sooraj N P, Athira K, M. S. Pillai, Jaishanker R

**Affiliations:** 1 C V Raman Laboratory of Ecological Informatics, Indian Institute of Information Technology and Management – Kerala, Thiruvananthapuram, Kerala 695581 India; 2 School of Informatics, Kerala University of Digital Sciences, Innovation and Technology, Technopark Phase - IV, Trivandrum, Kerala 695317 India; 3 Cochin University of Science and Technology, Cochin, Kerala 682022 India; 4 School of Ecology and Environment Studies, Nalanda University, Rajgir, Bihar 803116 India

**Keywords:** Ecology, Biodiversity, Ecology, Biodiversity, Conservation biology

## Abstract

Anthrophony is an important determinant of habitat quality in the Anthropocene. Acoustic adaptation of birds at lower levels of anthrophony is known. However, threshold anthrophony, beyond which biophony starts decreasing, is less explored. Here, we present empirical results of the relationship between anthrophony and biophony in four terrestrial soundscapes. The constancy of the predicted threshold vector normalised anthropogenic power spectral density (~ 0.40 Watts/Hz) at all the study sites is intriguing. We propose the threshold value of anthropogenic power spectral density as an indicator of the avian acoustic tolerance level in the study sites. The findings pave the way to determine permissible sound levels within protected landscapes and directly contribute to conservation planning.

## Introduction

The prospects of characterising habitats and ecosystems using acoustic data have attracted researchers from various disciplines to acoustic ecology. Over the last few decades, there has been a groundswell of interest in using sound to describe and characterise ecosystems^1^. The meteoric rise in technological capabilities and the plummeting cost of associated hardware^2–4^ helped acoustic ecology permeate laboratories worldwide. Ongoing research in acoustic ecology can broadly be classified as studies focusing on the landscape (community) and species levels. While the former is confined predominantly to interpreting acoustic indices^5^, the latter leverages the analytical powers of the current technology wave^6,7^. Either way, the primary focus is to extract ecological information from sonic data. Acoustic ecology is built up on the premise that a time-stamped soundscape is a signature of a landscape.

An offshoot of landscape ecology^8^, acoustic ecology is weighed down by the plurality of views and the lack of physical theories of the macroecological significance of soundscape^9^. The weak consensus on the interpretation of acoustic indices stems from the prevalent plurality. Intrinsic characteristics of mechanical waves, their propagation, and obstructions in terrestrial landscapes add to the complexity of acoustic ecology^10,11^. Notwithstanding the advances in field recording techniques that help circumvent some of the challenges posed by the mechanical nature of sound waves^12^, the reciprocating nature of communication^13^ of indicator species (here, birds)^14–18^ increases the probability to miss capturing the vocalisation of all bird species present during acoustic data collection in a terrestrial landscape. A lack of scientific consensus on the duration and periodicity of acoustic data measurement also impedes progress in the domain. While strong reasons are put forward as arguments favouring extended and continuous sonic recording^19,20^, the counter-arguments are equally strong and multidimensional^21,22^.

The plurality of perspectives in acoustic ecology posits an absorbing canvas. It remains ambiguous whether biophonic heterogeneity can be ascribed to the diversity of vocalising species or the community diversity^10^. Despite the different perceptions, the scientific community of acoustic ecologists is unanimous in advocating the utility of acoustic data in biodiversity studies. The domain is gaining currency as a travelator, which we can ill-afford to overlook for timely biodiversity assessment and conservation^23,24^. The effectiveness and adoption of acoustic metrics for biodiversity monitoring hinge on unravelling the underlying physical theories and developing mathematical constructs that aid objective conservation planning^25^. Formulating nomothetic theories in acoustic ecology is a need of the hour^26,27^. Soundscape dynamics, with its reflexive and evolutionary adaptations, posits the biggest hurdles in developing theories in acoustic ecology. Despite the thin ice on which acoustic ecological theories get constructed, the looming global biodiversity crisis leaves no option but to design innovative studies that lead to pragmatic results. The effectiveness of future conservation interventions hinges on translational research.

Here, we present empirical results of the affiliation between vector normalised power spectral density of anthrophony (0–2 kHz) and avian biophony (2–8 kHz) within a wildlife sanctuary, an urban park, and two sacred groves in Kerala, India. The results transform as permissible sound thresholds in terrestrial landscapes for effective conservation.

## Result and discussion

### Relationship between *α* and *β*

**Figure 1:**
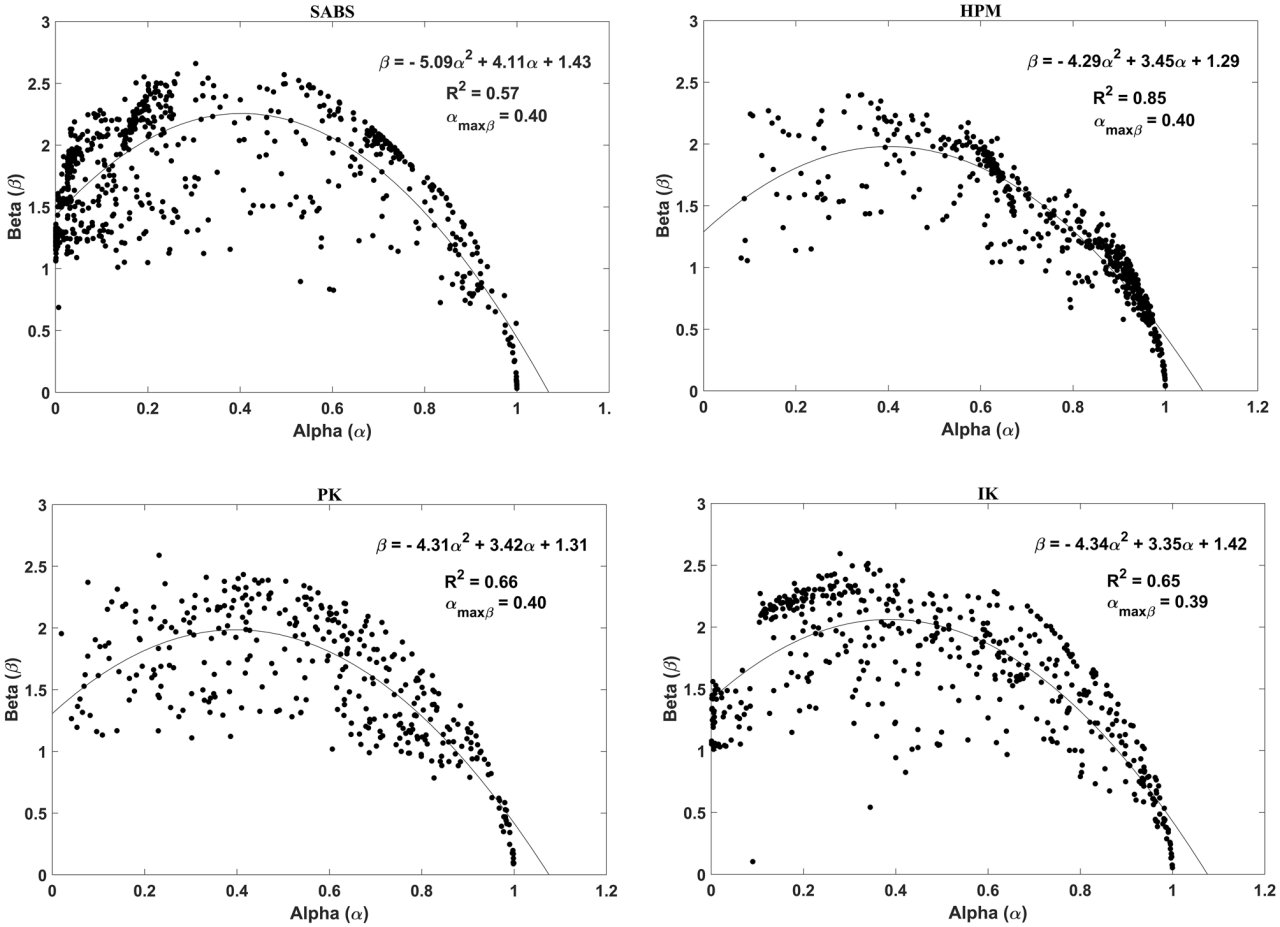
*α–β* regression model of the four sites *SABS*, *HPM*, *PK*, and *IK*.

The normalized power spectral density of the anthrophony (*α*) and biophony (*β*) components derived from the sonic database at *SABS*, *HPM*, *PK*, and *IK* are presented in Supplementary Table 1. The regression analysis of the *α* and *β* in each soundscape fits into concave-down quadratic regression models (Fig. 1). Table 1 summarises the quadratic regression models at the four sites. *HPM*, *PK*, and *IK* reveal matching patterns with comparable quadratic equation coefficients and standard deviations (Table 1). The consistently low standard deviation indicates fewer Power Spectral Density (*PSD*) fluctuations in the quadratic fit. While the regression model of *SABS* was slightly different from the others, the overall trends of the sites are comparable. The statistical significance (*p*-value < 0.05) and high *R*^2^ values (0.57, 0.85, 0.66, 0.65 for *SABS*, *HPM*, *PK*, and *IK*, respectively) of datasets describe sonic powers are fit for the regression models.

**Table 1:** Summary of the* α–β* regression model at the four sites.

Study site	*α*	*α*^2^	(Constant)	*R*^2^	*β*_*max*_ (Watts/Hz)	*α*_*maxβ*_ (Watts/Hz)
Salim Ali Bird Sanctuary (*SABS*)	4.11 (± 0.16)	− 5.1 (± 0.17)	1.43 (± 0.02)	0.57	2.26	0.40
Hill Palace Museum (*HPM*)	3.45 (± 0.22)	− 4.29 (± 0.17)	1.29 (± 0.06)	0.85	1.98	0.40
Poyil Kavu (*PK*)	3.42 (± 0.28)	− 4.31 (± 0.25)	1.31 (± 0.07)	0.66	1.99	0.40
Iringole Kavu (*IK*)	3.35 (± 0.20)	− 4.34 (± 0.19)	1.42 (± 0.04)	0.65	2.06	0.39

The *α*_*maxβ*_ and *β*_*max*_ of the fitted curves are given in Table 1. *α*_*maxβ*_ values describe the changing behaviour of soundscapes across anthrophonic (*α)* and biophonic (*β)* components. The highest *β*_*max*_ (2.26 Watts/Hz) was observed at *SABS*. The four sites showed almost identical values *α*_*maxβ*_ (0.39–0.40 Watts/Hz).

### Ecological significance of *α*_*maxβ*_

*PSD* represents average sonic power during a specific time in a certain frequency range. It is a physical measure of information that leads to understanding the spatio-temporal dynamics of soundscapes. Mechanical and biological sounds are prevalent between 1–2 and 2–8 kHz, respectively^28,29^. The frequency ranges are divided into 1 kHz frequency bins, and *α* and *β* estimate the vector normalised power spectral density of the anthrophony and biophony components by the sum of the power in these frequency bins. Since the prevalent anthrophonic range contains only one bin (1–2 kHz), the maximum *α* yields a power of 1 W/Hz^30^. This explains the convergence of *α* at 1 in Fig. 1. The magnitude of *β* represents the intensity of the biophony and thus reveals specific characteristics of vocal organisms in a soundscape.

The Ordinary Least Square regression analysis of *α* and *β* components across all the sites fits into concave down quadratic functions. Biophony (*β*) increases with increasing anthrophony (*α*) to a maximum before decreasing. The empirical results presented here correspond well with the Lombard effect^31,32^. The positive relationship of *α* and *β* at lower levels of *α* at all sites (Fig. 1) explicates the biophonic adaptive resilience of birds to changing soundscapes. However, the biophonic resilience collapses to zero after *β*_*max*_ with increasing *α* (Fig. 1). Higher *β* indicates the intensified bird vocalisations at the study sites and their presence.

Anthrophonic level in a landscape (*α*) is a proxy for the degree of disturbance and stress to non-human vocalising species. The notion of *α*_*maxβ*_ introduced in this paper is identical to the point corresponding to the vertex in Functional Calculus. It is the critical point where a curve changes direction from increasing to decreasing. Geometrically *α*_*maxβ*_ is the point at which the axis of symmetry through the vertex of the quadratic curve cuts the x-axis (*α*). The identical *α*_*maxβ*_ observed at all the sites (Table 1) open the prospect of defining acoustic limits in protected terrestrial landscapes. Elevated anthrophonic levels disturb indicator species like birds. They either become alarmed and silent or move to another soundscape with lower anthrophony^22,33^. Constant *α*_*maxβ*_ at the four sites point to similarities in their soundscapes. We presume *α*_*maxβ*_ to be dependent on geography. All sites in the present study are located in the tropical monsoon region. We put forth *α*_*maxβ*_ as a metric to denote the anthrophonic tolerance level of birds at the study sites.

Although several indices are available to study the presence and diversity of acoustic communities^19,34,35^, we are yet to arrive at a standard metric to denote the sonic characteristics of natural soundscapes. We propose estimating *α*_*maxβ*_ from the *α*–*β* regression model of the soundscapes as a pragmatic way to define the threshold anthrophonic sound in protected landscapes. The estimated *α*_*maxβ*_ of protected landscapes (soundscapes) provides a metric that can be used as the permissible threshold of anthrophony in protected landscapes. However, the validity and utility of *α* and *β* components of the soundscapes need to be further explored across multiple habitats by understanding the relationships between acoustic indices, biodiversity, and anthropogenic activities through proper habitat assessment. If so, the* α*–*β* regression model of soundscape will be a useful characteristic of the terrestrial landscape. Accordingly, it acts as a good surrogate that can be used to monitor habitat quality and taken as a baseline measure for landscape conservation planning. *α*–*β* regression models and* α*_*maxβ*_ are independent of the vocalization of individual species. While the Lombard effect in Aves can be considered as reflexive-adaptive^36^, the cumulative effect of persistent background anthrophony is known to shape bird sounds^37–40^. Consequently, baseline *α*–*β* regression models and* α*_*maxβ*_ can ingeniously be used to study drift in terrestrial soundscape, if any, over time.

Integrating the *α*–*β* regression models and *α*_*maxβ*_ into cost-effective conservation technologies opens pathways to quickly understand the anthrophonic tolerance level of birds, acoustic community structure, and their changes in response to environmental changes and anthropogenic activity. It encourages traditional bio-acoustics and biodiversity researchers unfamiliar with intensive acoustic-computational methods to arrive at recommendations for conservation policies. Though acoustic monitoring presents several advantages, as previously discussed, it also has certain limitations. References^10,41^, particularly at the community-level studies. Comprehensive field recordings, as well as the retrieval and management of data, require the substantial deployment of acoustic sensors and the corresponding hardware, leading to higher costs. Systematic ground-truthing of acoustics indices and measures against ecosystem parameters across multiple habitats is inevitable^42^ to explicitly develop new analyses and techniques inferring meaningful ecological information^43^ Given the challenges mentioned above, deploying the proposed *α*–*β* regression models and αmaxβ for landscape conservation is currently challenging. However, our findings are transformative, rendering acoustic ecology exigent in conservation efforts. Validating this finding across diverse regions with varying spatial densities of acoustic sensors and temporal frequencies will possess an improved method for monitoring landscapes in the future.

## Materials and methods

### Study area

Acoustic data were collected from Salim Ali Bird Sanctuary (*SABS*), Hill Palace Museum (*HPM*), Poyil Kavu (*PK*), and Irigole Kavu (*IK*) in Kerala, India, in 2018 and 2019, respectively. *SABS* is an International Bird Area (IBA)^44^ located along the bank of the Periyar River spread over 25.16 km^2^ and lies between 10° 7′ and 11° N latitudes and 76° 40′ and 76° 45′ E longitudes. Of the 284 bird species observed at *SABS*, 03 are vulnerable, 08 are near-threatened, and 11 are endemic. *SABS* also provides seasonal refuge to 72 migratory bird species.

*HPM* is an urban park spread across 22.8 ha, bound by 9° 57′ 10.27′′ N latitude and 76° 21′ 49.94′′ E longitude in the Ernakulam district. *PK* and *IK* are sacred groves in urban settings. *PK* is located at 11° 24′ 35.05′′ N latitude and 75° 42′ 58.55′′ E longitude in the Kozhikode district and sandwiched in 12 ha of land between the National Highway (NH 66) and Kappad beach. *IK* is situated at 10° 6′ 30.95′′ N latitudes and 76° 30′ 1.81′′ E longitude in the Ernakulam district and is spread across 20 ha.

### Acoustic data collection

Acoustic recording was carried out at the study sites following standard protocol^45^. We focused on avian sounds, as they are indicator species^27,46^. As most bird species within the study sites were diurnal^23^, only daytime acoustic recording over a 12 h time window from 6.00 AM to 6.00 PM was carried out. All recordings were carried out at preselected locations within the study sites. Ten sound clips of 1 min each were recorded every hour, and their mean was taken as the representative acoustic sample of the respective hour. This processed data of 1-min duration is sufficient to analyse and provide potentially rich sources of ecological information about the abundance, distribution, and behaviour of avian species^47^. Acoustic data of *SABS* were recorded on 19 April, 07 September, 11 December 2018, 18 April 2019, 09 September 2019, and 10 December 2019 at about > 500 m away from the river. Acoustic data of *HPM* and *IK* were collected for the same duration one day before and after that of the measurement at *SABS* in April, December 2018, and 2019, respectively. An identical framework was used to record the acoustic data of *PK* on 14 December 2018 and 21 April 2019. All the recordings at HPM, PK, and IK were carried out at the interior locations. The unitary dates were representative samples of summer, post-monsoon, and winter at the study sites. The acoustic measurements were carried out using Marantz PMD 661 MK III sonic recorder with an omnidirectional boundary microphone at 44.1 kHz/16-bit sampling rate. Acoustic data was stored in .wav format as signals in two channels (left and right).

### Data analysis

Acoustic Data from each site was pooled separately for analysis. The Welch Power Spectral Density (*PSD*) (Watts/Hz) in the frequency range between 1–2 kHz (*α*), and 2–8 kHz (*β*), of *SABS*, *HPM*, *PK*, and *IK* soundscapes at the two time periods were extracted as the average of the left and right channels using Tune R®^48^ and ndsi() function in soundecology®^49^ packages in R v.3.1.2^50^.

The relationship between *α* and *β* was estimated using Ordinary Least Square regression. We determined the *α*_*maxβ*_, where the fitted *α* function changes from increasing to decreasing (points correspond to maximum *β*,* β*_*max*_) by calculating the axis of symmetry of a quadratic function:

1 $${\alpha }_{max\beta }=\frac{-b}{a},$$

where *a* and *b* are the coefficients of the quadratic fit function $\beta (\alpha )=a{\alpha }^{2}+b\alpha +c$.

### Supplementary Information

Supplementary Table 1.

## Data Availability

All data generated or analysed during this study are included in this published article (and its Supplementary Information files).

## References

[1] Eldridge, A. & Kiefer, C. Toward a synthetic acoustic ecology: Sonically situated, evolutionary agent based models of the acoustic niche hypothesis. In *Proc. Artificial Life Conference* 296–303 (2020).

[2] Truskinger, A., Cottman-Fields, M., Eichinski, P., Towsey, M. & Roe, P. Practical analysis of big acoustic sensor data for environmental monitoring. In *Proceedings—4th IEEE International Conference on Big Data and Cloud Computing, BDCloud 2014 with the 7th IEEE International Conference on Social Computing and Networking, SocialCom 2014 and the 4th International Conference on Sustainable Computing and C* 91–98. 10.1109/BDCloud.2014.29 (2014).

[3] Almeira J, Guecha S (2019). Dominant power spectrums as a tool to establish an ecoacoustic baseline in a premontane moist forest. Landsc. Ecol. Eng..

[4] Farina A, Gage SH (2017). Ecoacoustics: The Ecological Role of Sounds.

[5] Sueur J, Farina A, Gasc A, Pieretti N, Pavoine S (2014). Acoustic indices for biodiversity assessment and landscape investigation. Acta Acoust. United Acust..

[6] Bianco MJ (2019). Machine learning in acoustics: Theory and applications. J. Acoust. Soc. Am..

[7] Stowell D, Petrusková T, Šálek M, Linhart P (2019). Automatic acoustic identification of individuals in multiple species: Improving identification across recording conditions. J. R. Soc. Interface.

[8] Turner MG, Gardner RH (2015). Landscape ecology in theory and practice. Pattern Process..

[9] Sueur J, Pavoine S, Hamerlynck O, Duvail S (2008). Rapid acoustic survey for biodiversity appraisal. PLoS ONE.

[10] Gibb R, Browning E, Glover-Kapfer P, Jones KE (2019). Emerging opportunities and challenges for passive acoustics in ecological assessment and monitoring. Methods Ecol. Evol..

[11] Wiley RH, Richards DG, Wiley RH, Richards DG (1982). Adaptations for acoustic communication in birds: Sound transmission and signal detection. Acoustic Communication in Birds.

[12] Merchant ND (2015). Measuring acoustic habitats. Methods Ecol. Evol..

[13] Müller JJA, Massen JJM, Bugnyar T, Osvath M (2017). Ravens remember the nature of a single reciprocal interaction sequence over 2 days and even after a month. Anim. Behav..

[14] Hostetler M, Holling CS (2000). Detecting the scales at which birds respond to structure in urban landscapes. Urban Ecosyst..

[15] Bolger DT, Scott TA, Rotenberry JT (2001). Use of corridor-like landscape structures by bird and small mammal species. Biol. Conserv..

[16] Fernández-Juricic E (2004). Spatial and temporal analysis of the distribution of forest specialists in an urban-fragmented landscape (Madrid, Spain): Implications for local and regional bird conservation. Landsc. Urban Plan..

[17] Gregory R (2003). Using birds as indicators of biodiversity. Ornis Hungarica.

[18] Mekonen S (2017). Birds as biodiversity and environmental indicator. Adv. Life Sci. Technol..

[19] Bradfer-Lawrence T (2019). Guidelines for the use of acoustic indices in environmental research. Methods Ecol. Evol..

[20] Towsey M (2014). Visualization of long-duration acoustic recordings of the environment. Procedia Comput. Sci..

[21] Hubert J, Neo YY, Winter HV, Slabbekoorn H (2020). The role of ambient sound levels, signal-to-noise ratio, and stimulus pulse rate on behavioural disturbance of seabass in a net pen. Behav. Process..

[22] Barber JR, Crooks KR, Fristrup KM (2010). The costs of chronic noise exposure for terrestrial organisms. Trends Ecol. Evol..

[23] Rajan SC, Athira K, Jaishanker R, Sooraj NP, Sarojkumar V (2019). Rapid assessment of biodiversity using acoustic indices. Biodivers. Conserv..

[24] Eldridge A (2018). Sounding out ecoacoustic metrics: Avian species richness is predicted by acoustic indices in temperate but not tropical habitats. Ecol. Indic..

[25] Farina A, Reid V (2020). The ecological role of sound in terrestrial and aquatic landscape: Theories, methods and applications of ecoacoustics. Biodiversity.

[26] Pijanowski BC (2011). Soundscape ecology: The science of sound in the landscape. Bioscience.

[27] Gasc A, Francomano D, Dunning JB, Pijanowski BC (2017). Future directions for soundscape ecology: The importance of ornithological contributions. Auk.

[28] Gage S, Napoletano B (2004). Envirosonics Equipment and Operations Manual.

[29] Gage SH, Napoletano BM, Cooper MC (2001). Assessment of ecosystem biodiversity by acoustic diversity indices. J. Acoust. Soc. Am..

[30] Kasten EP, Gage SH, Fox J, Joo W (2012). The remote environmental assessment laboratory’s acoustic library: An archive for studying soundscape ecology. Ecol. Inform..

[31] Brumm H, Naguib M, Brumm H, Naguib M (2009). Environmental acoustics and the evolution of bird song. Advances in the Study of Behavior.

[32] Schuster S, Zollinger SA, Lesku JA, Brumm H (2012). On the evolution of noise-dependent vocal plasticity in birds. Biol. Lett..

[33] Kuehne LM, Padgham BL, Olden JD (2013). The soundscapes of lakes across an urbanization gradient. PLoS ONE.

[34] Fairbrass AJ, Rennett P, Williams C, Titheridge H, Jones KE (2017). Biases of acoustic indices measuring biodiversity in urban areas. Ecol. Indic..

[35] Farina A, Righini R, Fuller S, Li P, Pavan G (2021). Acoustic complexity indices reveal the acoustic communities of the old-growth Mediterranean forest of Sasso Fratino Integral Natural Reserve (Central Italy). Ecol. Indic..

[36] Brumm H, Todt D (2002). Noise-dependent song amplitude regulation in a territorial songbird. Anim. Behav..

[37] Brumm H (2004). The impact of environmental noise on song amplitude in a territorial bird. J. Anim. Ecol..

[38] To AWY, Dingle C, Collins SA (2021). Multiple constraints on urban bird communication: Both abiotic and biotic noise shape songs in cities. Behav. Ecol..

[39] Olson CR, Fernandez-Peters M, Portfors CV, Mello CV (2018). Black Jacobin hummingbirds vocalize above the known hearing range of birds. Curr. Biol..

[40] Clark ML (2023). The effect of soundscape composition on bird vocalization classification in a citizen science biodiversity monitoring project. Ecol. Inform..

[41] Sethi SS (2023). Limits to the accurate and generalizable use of soundscapes to monitor biodiversity. Nat. Ecol. Evol..

[42] Sueur J, Farina A (2015). Ecoacoustics: The ecological investigation and interpretation of environmental sound. Biosemiotics.

[43] Eldridge A, Casey M, Moscoso P, Peck M (2016). A new method for ecoacoustics? Toward the extraction and evaluation of ecologically-meaningful soundscape components using sparse coding methods. PeerJ.

[44] BirdLife International. *Important Bird Areas Factsheet: Thattekkad Wildlife Sanctuary*. http://www.birdlife.org (2019).

[45] Browning E, Gibb R, Glover-Kapfer P, Jones KE (2017). Passive acoustic monitoring in ecology and conservation. WWF Conserv. Technol. Ser..

[46] Wimmer J, Towsey M, Roe P, Williamson I (2013). Sampling environmental acoustic recordings to determine bird species richness. Ecol. Appl..

[47] Bradfer-Lawrence T, Bunnefeld N, Gardner N, Willis SG, Dent DH (2020). Rapid assessment of avian species richness and abundance using acoustic indices. Ecol. Indic..

[48] Uwe Ligges. *TuneR: Analysis of Music*. http://www.ci.tuwien.ac.at/Conferences/useR-2004/abstracts/Ligges.pdf (2004).

[49] Villanueva-Rivera, L. J. & Pijanowski, B. C. *Package ‘Soundecology’*. http://ljvillanueva.github.io/soundecology/ (CRAN, 2016).

[50] R Core Team. *R: A Language and Environment for Statistical Computing* (2014).

