# Supplementary material for: Threshold of anthropogenic sound levels within protected landscapes in Kerala, India, for avian habitat quality and conservation
Source: Sci Rep. 2024 Feb 1;14:2701. doi: 10.1038/s41598-024-53153-6 (PMC10834939; doi:10.1038/s41598-024-53153-6)
Supplement: Supplementary file 1 — Supplementary Table 1. [file 41598_2024_53153_MOESM1_ESM.docx]

# Threshold of anthropogenic sound levels within protected landscapes in Kerala, India, for avian habitat quality and conservation

# Sajeev C Rajan^1,4^, Vishnu M^1,2^, Ahalya Mitra^2^, Sooraj N.P^2^, Athira K^2^, M S Pillai^2^ and R. Jaishanker^1,2,3,*^

# C V Raman Laboratory of Ecological Informatics,

# Indian Institute of Information Technology and Management – Kerala,

# Thiruvananthapuram, Kerala – 695581, India.

1. School of Informatics

Kerala University of Digital Sciences Innovation and Technology

Technopark Phase - IV, Trivandrum, Kerala – 695317, India.

1. School of Ecology and Environment Studies, Nalanda University, Rajgir, Bihar – 803116, India.
2. Cochin University of Science and Technology, Cochin, Kerala – 682022, India

# *Corresponding author: jrnair@duk.ac.in

# ORCID: 0000-0002-6570-9411

**Supplementary Table 1**: Vector normalised power spectral density of Anthrophony **(***α*) and Biophony **(***β***)** values at Salim Ali Bird Sanctuary (SABS), Irigole Kavu (IK), Hill Palace Museum (HPM), and Poyil Kavu (PK), in Kerala, India.

| **Sl.No** | **Site** | **Year** | **Month** | **Anthrophony (*α*) (Watts/Hz)** | **Biophony (*β*)**  **(Watts/Hz)** |
| --- | --- | --- | --- | --- | --- |
| 1 | SABS | 2018 | April | 0.0092 | 1.2119 |
| 2 | SABS | 2018 | April | 0.0180 | 1.2346 |
| 3 | SABS | 2018 | April | 0.0142 | 1.2669 |
| 4 | SABS | 2018 | April | 0.0383 | 1.2869 |
| 5 | SABS | 2018 | April | 0.0406 | 1.2850 |
| 6 | SABS | 2018 | April | 0.0370 | 1.3181 |
| 7 | SABS | 2018 | April | 0.0154 | 1.2970 |
| 8 | SABS | 2018 | April | 0.0317 | 1.2417 |
| 9 | SABS | 2018 | April | 0.0244 | 1.2456 |
| 10 | SABS | 2018 | April | 0.0176 | 1.2477 |
| 11 | SABS | 2018 | April | 0.0057 | 1.2060 |
| 12 | SABS | 2018 | April | 0.0047 | 1.1819 |
| 13 | SABS | 2018 | April | 0.0051 | 1.2150 |
| 14 | SABS | 2018 | April | 0.0087 | 1.3097 |
| 15 | SABS | 2018 | April | 0.0046 | 1.2850 |
| 16 | SABS | 2018 | April | 0.0059 | 1.3184 |
| 17 | SABS | 2018 | April | 0.0039 | 1.3230 |
| 18 | SABS | 2018 | April | 0.0223 | 1.2965 |
| 19 | SABS | 2018 | April | 0.0719 | 1.3292 |
| 20 | SABS | 2018 | April | 0.0090 | 1.2886 |
| 21 | SABS | 2018 | April | 0.0010 | 1.3088 |
| 22 | SABS | 2018 | April | 0.0040 | 1.3283 |
| 23 | SABS | 2018 | April | 0.0024 | 1.2595 |
| 24 | SABS | 2018 | April | 0.0018 | 1.2594 |
| 25 | SABS | 2018 | April | 0.0012 | 1.2265 |
| 26 | SABS | 2018 | April | 0.0061 | 1.5651 |
| 27 | SABS | 2018 | April | 0.0044 | 1.5015 |
| 28 | SABS | 2018 | April | 0.0173 | 1.9063 |
| 29 | SABS | 2018 | April | 0.0007 | 1.2935 |
| 30 | SABS | 2018 | April | 0.0003 | 1.2215 |
| 31 | SABS | 2018 | April | 0.0012 | 1.3181 |
| 32 | SABS | 2018 | April | 0.0018 | 1.2874 |
| 33 | SABS | 2018 | April | 0.0007 | 1.3599 |
| 34 | SABS | 2018 | April | 0.0012 | 1.3166 |
| 35 | SABS | 2018 | April | 0.0003 | 1.1513 |
| 36 | SABS | 2018 | April | 0.0005 | 1.1975 |
| 37 | SABS | 2018 | April | 0.0004 | 1.2479 |
| 38 | SABS | 2018 | April | 0.0006 | 1.1594 |
| 39 | SABS | 2018 | April | 0.0003 | 1.2818 |
| 40 | SABS | 2018 | April | 0.0004 | 1.3157 |
| 41 | SABS | 2018 | April | 0.0004 | 1.2669 |
| 42 | SABS | 2018 | April | 0.0004 | 1.3205 |
| 43 | SABS | 2018 | April | 0.0005 | 1.2615 |
| 44 | SABS | 2018 | April | 0.0004 | 1.3148 |
| 45 | SABS | 2018 | April | 0.0004 | 1.3007 |
| 46 | SABS | 2018 | April | 0.0004 | 1.3111 |
| 47 | SABS | 2018 | April | 0.0004 | 1.3336 |
| 48 | SABS | 2018 | April | 0.0004 | 1.2047 |
| 49 | SABS | 2018 | April | 0.2006 | 2.0716 |
| 50 | SABS | 2018 | April | 0.0093 | 1.5272 |
| 51 | SABS | 2018 | April | 0.2295 | 2.2285 |
| 52 | SABS | 2018 | April | 0.0321 | 1.5000 |
| 53 | SABS | 2018 | April | 0.1817 | 1.2446 |
| 54 | SABS | 2018 | April | 0.1998 | 1.0496 |
| 55 | SABS | 2018 | April | 0.1492 | 1.0523 |
| 56 | SABS | 2018 | April | 0.0458 | 1.0900 |
| 57 | SABS | 2018 | April | 0.1081 | 1.1838 |
| 58 | SABS | 2018 | April | 0.1140 | 1.2998 |
| 59 | SABS | 2018 | April | 0.2009 | 2.1844 |
| 60 | SABS | 2018 | April | 0.2393 | 1.9950 |
| 61 | SABS | 2018 | April | 0.2117 | 2.1457 |
| 62 | SABS | 2018 | April | 0.2091 | 1.9614 |
| 63 | SABS | 2018 | April | 0.4418 | 1.9066 |
| 64 | SABS | 2018 | April | 0.1328 | 2.1295 |
| 65 | SABS | 2018 | April | 0.2193 | 1.9338 |
| 66 | SABS | 2018 | April | 0.1481 | 1.9935 |
| 67 | SABS | 2018 | April | 0.0044 | 1.4333 |
| 68 | SABS | 2018 | April | 0.0645 | 1.7223 |
| 69 | SABS | 2018 | April | 0.1121 | 1.6778 |
| 70 | SABS | 2018 | April | 0.1302 | 1.6679 |
| 71 | SABS | 2018 | April | 0.1095 | 2.0710 |
| 72 | SABS | 2018 | April | 0.6134 | 1.6694 |
| 73 | SABS | 2018 | April | 0.6577 | 1.2267 |
| 74 | SABS | 2018 | April | 0.0925 | 1.5512 |
| 75 | SABS | 2018 | April | 0.0925 | 1.5512 |
| 76 | SABS | 2018 | April | 0.1506 | 1.6468 |
| 77 | SABS | 2018 | April | 0.0947 | 1.5833 |
| 78 | SABS | 2018 | April | 0.0137 | 1.6300 |
| 79 | SABS | 2018 | April | 0.0137 | 1.7652 |
| 80 | SABS | 2018 | April | 0.0182 | 1.6493 |
| 81 | SABS | 2018 | April | 0.0714 | 1.4907 |
| 82 | SABS | 2018 | April | 0.2094 | 1.4876 |
| 83 | SABS | 2018 | April | 0.0341 | 1.5550 |
| 84 | SABS | 2018 | April | 0.0134 | 1.5341 |
| 85 | SABS | 2018 | April | 0.0318 | 1.5798 |
| 86 | SABS | 2018 | April | 0.0356 | 1.8000 |
| 87 | SABS | 2018 | April | 0.0417 | 1.5024 |
| 88 | SABS | 2018 | April | 0.0338 | 2.0794 |
| 89 | SABS | 2018 | April | 0.1171 | 1.7551 |
| 90 | SABS | 2018 | April | 0.1261 | 1.7542 |
| 91 | SABS | 2018 | April | 0.7830 | 1.2075 |
| 92 | SABS | 2018 | April | 0.0916 | 1.8612 |
| 93 | SABS | 2018 | April | 0.0408 | 1.6343 |
| 94 | SABS | 2018 | April | 0.0167 | 1.5684 |
| 95 | SABS | 2018 | April | 0.0186 | 1.8338 |
| 96 | SABS | 2018 | April | 0.0172 | 1.5531 |
| 97 | SABS | 2018 | April | 0.0129 | 1.4988 |
| 98 | SABS | 2018 | April | 0.0269 | 1.5460 |
| 99 | SABS | 2018 | April | 0.0003 | 1.2095 |
| 100 | SABS | 2018 | April | 0.0003 | 1.1870 |
| 101 | SABS | 2018 | April | 0.0003 | 1.1887 |
| 102 | SABS | 2018 | April | 0.0003 | 1.1811 |
| 103 | SABS | 2018 | April | 0.0003 | 1.2306 |
| 104 | SABS | 2018 | April | 0.0003 | 1.2504 |
| 105 | SABS | 2018 | April | 0.0005 | 1.2211 |
| 106 | SABS | 2018 | April | 0.0039 | 1.2606 |
| 107 | SABS | 2018 | April | 0.0049 | 1.2499 |
| 108 | SABS | 2018 | April | 0.0030 | 1.2349 |
| 109 | SABS | 2018 | April | 0.0394 | 1.4303 |
| 110 | SABS | 2018 | April | 0.0260 | 1.4075 |
| 111 | SABS | 2018 | April | 0.0794 | 1.3739 |
| 112 | SABS | 2018 | April | 0.1312 | 1.5224 |
| 113 | SABS | 2018 | April | 0.0936 | 1.5137 |
| 114 | SABS | 2018 | April | 0.0194 | 1.2909 |
| 115 | SABS | 2018 | April | 0.4070 | 1.6360 |
| 116 | SABS | 2018 | April | 0.8005 | 1.5399 |
| 117 | SABS | 2018 | April | 0.5918 | 1.7067 |
| 118 | SABS | 2018 | April | 0.0024 | 1.1963 |
| 119 | SABS | 2018 | April | 0.0029 | 1.1877 |
| 120 | SABS | 2018 | April | 0.0020 | 1.1593 |
| 121 | SABS | 2018 | April | 0.0017 | 1.1592 |
| 122 | SABS | 2018 | April | 0.0010 | 1.0878 |
| 123 | SABS | 2018 | April | 0.0005 | 1.0663 |
| 124 | SABS | 2018 | April | 0.0005 | 1.0636 |
| 125 | SABS | 2018 | April | 0.0006 | 1.0706 |
| 126 | SABS | 2018 | April | 0.0011 | 1.1238 |
| 127 | SABS | 2018 | April | 0.0018 | 1.1954 |
| 128 | SABS | 2019 | April | 0.1590 | 2.1094 |
| 129 | SABS | 2019 | April | 0.1650 | 2.1376 |
| 130 | SABS | 2019 | April | 0.1736 | 2.2195 |
| 131 | SABS | 2019 | April | 0.1759 | 2.1996 |
| 132 | SABS | 2019 | April | 0.1461 | 1.9987 |
| 133 | SABS | 2019 | April | 0.1494 | 2.0248 |
| 134 | SABS | 2019 | April | 0.1467 | 2.1089 |
| 135 | SABS | 2019 | April | 0.1400 | 2.0822 |
| 136 | SABS | 2019 | April | 0.1467 | 2.1077 |
| 137 | SABS | 2019 | April | 0.1524 | 2.1183 |
| 138 | SABS | 2019 | April | 0.1906 | 2.2079 |
| 139 | SABS | 2019 | April | 0.1846 | 2.2024 |
| 140 | SABS | 2019 | April | 0.1993 | 2.1985 |
| 141 | SABS | 2019 | April | 0.1715 | 2.2006 |
| 142 | SABS | 2019 | April | 0.1708 | 2.2275 |
| 143 | SABS | 2019 | April | 0.1838 | 2.2673 |
| 144 | SABS | 2019 | April | 0.2047 | 2.1771 |
| 145 | SABS | 2019 | April | 0.2011 | 2.4959 |
| 146 | SABS | 2019 | April | 0.1817 | 2.3279 |
| 147 | SABS | 2019 | April | 0.1890 | 2.3496 |
| 148 | SABS | 2019 | April | 0.2018 | 2.4385 |
| 149 | SABS | 2019 | April | 0.2222 | 2.4334 |
| 150 | SABS | 2019 | April | 0.2272 | 2.4001 |
| 151 | SABS | 2019 | April | 0.1972 | 2.3864 |
| 152 | SABS | 2019 | April | 0.2164 | 2.4689 |
| 153 | SABS | 2019 | April | 0.4670 | 2.2785 |
| 154 | SABS | 2019 | April | 0.2167 | 2.4272 |
| 155 | SABS | 2019 | April | 0.2186 | 2.5034 |
| 156 | SABS | 2019 | April | 0.1601 | 2.2031 |
| 157 | SABS | 2019 | April | 0.1675 | 2.1704 |
| 158 | SABS | 2019 | April | 0.1595 | 2.1161 |
| 159 | SABS | 2019 | April | 0.1606 | 2.1974 |
| 160 | SABS | 2019 | April | 0.1600 | 2.1981 |
| 161 | SABS | 2019 | April | 0.1621 | 2.1817 |
| 162 | SABS | 2019 | April | 0.1693 | 2.2168 |
| 163 | SABS | 2019 | April | 0.1626 | 2.1751 |
| 164 | SABS | 2019 | April | 0.1722 | 2.1814 |
| 165 | SABS | 2019 | April | 0.1711 | 2.1340 |
| 166 | SABS | 2019 | April | 0.1629 | 2.1598 |
| 167 | SABS | 2019 | April | 0.1611 | 2.1532 |
| 168 | SABS | 2019 | April | 0.1525 | 2.0613 |
| 169 | SABS | 2019 | April | 0.1612 | 2.1024 |
| 170 | SABS | 2019 | April | 0.1591 | 2.1083 |
| 171 | SABS | 2019 | April | 0.1490 | 2.0808 |
| 172 | SABS | 2019 | April | 0.1504 | 2.0427 |
| 173 | SABS | 2019 | April | 0.1519 | 2.0935 |
| 174 | SABS | 2019 | April | 0.1549 | 2.1115 |
| 175 | SABS | 2019 | April | 0.1519 | 2.0482 |
| 176 | SABS | 2019 | April | 0.2656 | 2.0033 |
| 177 | SABS | 2019 | April | 0.1697 | 2.2404 |
| 178 | SABS | 2019 | April | 0.1727 | 2.2546 |
| 179 | SABS | 2019 | April | 0.1735 | 2.1817 |
| 180 | SABS | 2019 | April | 0.1724 | 2.2422 |
| 181 | SABS | 2019 | April | 0.1647 | 2.1046 |
| 182 | SABS | 2019 | April | 0.1698 | 2.1746 |
| 183 | SABS | 2019 | April | 0.1706 | 2.1608 |
| 184 | SABS | 2019 | April | 0.1626 | 2.1176 |
| 185 | SABS | 2019 | April | 0.1693 | 2.1387 |
| 186 | SABS | 2019 | April | 0.1610 | 2.0429 |
| 187 | SABS | 2019 | April | 0.1532 | 1.9815 |
| 188 | SABS | 2019 | April | 0.1532 | 1.9815 |
| 189 | SABS | 2019 | April | 0.1400 | 1.9264 |
| 190 | SABS | 2019 | April | 0.1232 | 1.8327 |
| 191 | SABS | 2019 | April | 0.1154 | 1.8006 |
| 192 | SABS | 2019 | April | 0.0917 | 1.6681 |
| 193 | SABS | 2019 | April | 0.1045 | 1.7445 |
| 194 | SABS | 2019 | April | 0.1321 | 1.9228 |
| 195 | SABS | 2019 | April | 0.1484 | 1.9744 |
| 196 | SABS | 2019 | April | 0.2009 | 2.4830 |
| 197 | SABS | 2019 | April | 0.2014 | 2.4494 |
| 198 | SABS | 2019 | April | 0.1880 | 2.2768 |
| 199 | SABS | 2019 | April | 0.2407 | 2.4779 |
| 200 | SABS | 2019 | April | 0.3038 | 2.3653 |
| 201 | SABS | 2019 | April | 0.3426 | 2.4809 |
| 202 | SABS | 2019 | April | 0.2469 | 2.4287 |
| 203 | SABS | 2019 | April | 0.3426 | 2.3845 |
| 204 | SABS | 2019 | April | 0.4005 | 2.3667 |
| 205 | SABS | 2019 | April | 0.3405 | 2.2318 |
| 206 | SABS | 2019 | April | 0.2142 | 2.3804 |
| 207 | SABS | 2019 | April | 0.2573 | 2.5131 |
| 208 | SABS | 2019 | April | 0.2024 | 2.2798 |
| 209 | SABS | 2019 | April | 0.2234 | 2.3282 |
| 210 | SABS | 2019 | April | 0.1941 | 2.2881 |
| 211 | SABS | 2019 | April | 0.1965 | 2.3612 |
| 212 | SABS | 2019 | April | 0.5374 | 2.0370 |
| 213 | SABS | 2019 | April | 0.2326 | 2.1271 |
| 214 | SABS | 2019 | April | 0.1925 | 2.3307 |
| 215 | SABS | 2019 | April | 0.1998 | 2.3116 |
| 216 | SABS | 2019 | April | 0.1942 | 2.1477 |
| 217 | SABS | 2019 | April | 0.1715 | 2.1372 |
| 218 | SABS | 2019 | April | 0.2386 | 2.4849 |
| 219 | SABS | 2019 | April | 0.2585 | 2.4981 |
| 220 | SABS | 2019 | April | 0.3194 | 2.3977 |
| 221 | SABS | 2019 | April | 0.2148 | 2.4291 |
| 222 | SABS | 2019 | April | 0.2282 | 2.5009 |
| 223 | SABS | 2019 | April | 0.3191 | 2.4001 |
| 224 | SABS | 2019 | April | 0.3686 | 2.3952 |
| 225 | SABS | 2019 | April | 0.2278 | 2.3069 |
| 226 | SABS | 2019 | April | 0.1680 | 2.2690 |
| 227 | SABS | 2019 | April | 0.1761 | 2.2907 |
| 228 | SABS | 2019 | April | 0.1800 | 2.2595 |
| 229 | SABS | 2019 | April | 0.1917 | 2.3163 |
| 230 | SABS | 2019 | April | 0.1948 | 2.3613 |
| 231 | SABS | 2019 | April | 0.1724 | 2.1299 |
| 232 | SABS | 2019 | April | 0.1947 | 2.3928 |
| 233 | SABS | 2019 | April | 0.1919 | 2.5522 |
| 234 | SABS | 2019 | April | 0.2059 | 2.4134 |
| 235 | SABS | 2019 | April | 0.2203 | 2.4980 |
| 236 | SABS | 2019 | April | 0.1903 | 2.3260 |
| 237 | SABS | 2019 | April | 0.2125 | 2.2261 |
| 238 | SABS | 2019 | April | 0.2393 | 2.3170 |
| 239 | SABS | 2019 | April | 0.2392 | 2.3664 |
| 240 | SABS | 2019 | April | 0.2490 | 2.2975 |
| 241 | SABS | 2019 | April | 0.2489 | 2.2741 |
| 242 | SABS | 2019 | April | 0.1585 | 1.9641 |
| 243 | SABS | 2019 | April | 0.2287 | 2.0828 |
| 244 | SABS | 2019 | April | 0.2351 | 2.0995 |
| 245 | SABS | 2019 | April | 0.9038 | 0.7933 |
| 246 | SABS | 2019 | April | 0.8972 | 0.7987 |
| 247 | SABS | 2019 | April | 0.9061 | 0.7973 |
| 248 | SABS | 2019 | April | 0.8818 | 0.8933 |
| 249 | SABS | 2019 | April | 0.8819 | 0.8447 |
| 250 | SABS | 2019 | April | 0.2648 | 1.4174 |
| 251 | SABS | 2019 | April | 0.5763 | 1.2485 |
| 252 | SABS | 2019 | April | 0.8999 | 0.7982 |
| 253 | SABS | 2019 | April | 0.8605 | 0.8735 |
| 254 | SABS | 2019 | April | 0.8367 | 0.9272 |
| 255 | SABS | 2018 | September | 0.3086 | 1.3128 |
| 256 | SABS | 2018 | September | 0.2846 | 1.2988 |
| 257 | SABS | 2018 | September | 0.1313 | 1.1492 |
| 258 | SABS | 2018 | September | 0.4896 | 1.7681 |
| 259 | SABS | 2018 | September | 0.2535 | 1.3738 |
| 260 | SABS | 2018 | September | 0.1760 | 1.7436 |
| 261 | SABS | 2018 | September | 0.2948 | 1.6050 |
| 262 | SABS | 2018 | September | 0.1235 | 1.4270 |
| 263 | SABS | 2018 | September | 0.0491 | 1.2364 |
| 264 | SABS | 2018 | September | 0.1260 | 1.4406 |
| 265 | SABS | 2018 | September | 0.1423 | 1.3457 |
| 266 | SABS | 2018 | September | 0.1600 | 1.4045 |
| 267 | SABS | 2018 | September | 0.1040 | 1.3110 |
| 268 | SABS | 2018 | September | 0.0964 | 1.3043 |
| 269 | SABS | 2018 | September | 0.0552 | 1.3515 |
| 270 | SABS | 2018 | September | 0.0799 | 1.3496 |
| 271 | SABS | 2018 | September | 0.0814 | 1.2445 |
| 272 | SABS | 2018 | September | 0.0624 | 1.3038 |
| 273 | SABS | 2018 | September | 0.0457 | 1.2167 |
| 274 | SABS | 2018 | September | 0.0525 | 1.2414 |
| 275 | SABS | 2018 | September | 0.8340 | 0.7261 |
| 276 | SABS | 2018 | September | 0.5755 | 1.1765 |
| 277 | SABS | 2018 | September | 0.3784 | 1.1571 |
| 278 | SABS | 2018 | September | 0.3210 | 1.1235 |
| 279 | SABS | 2018 | September | 0.2457 | 1.1258 |
| 280 | SABS | 2018 | September | 0.2477 | 1.1554 |
| 281 | SABS | 2018 | September | 0.1957 | 1.2999 |
| 282 | SABS | 2018 | September | 0.0331 | 1.5439 |
| 283 | SABS | 2018 | September | 0.0107 | 1.2915 |
| 284 | SABS | 2018 | September | 0.0067 | 0.6868 |
| 285 | SABS | 2018 | September | 0.0063 | 1.2931 |
| 286 | SABS | 2018 | September | 0.0046 | 1.2620 |
| 287 | SABS | 2018 | September | 0.0065 | 1.3116 |
| 288 | SABS | 2018 | September | 0.0070 | 1.2879 |
| 289 | SABS | 2018 | September | 0.0036 | 1.2668 |
| 290 | SABS | 2018 | September | 0.0031 | 1.2751 |
| 291 | SABS | 2018 | September | 0.0034 | 1.2784 |
| 292 | SABS | 2018 | September | 0.0040 | 1.2907 |
| 293 | SABS | 2018 | September | 0.0259 | 1.1253 |
| 294 | SABS | 2018 | September | 0.0295 | 1.1329 |
| 295 | SABS | 2018 | September | 0.1499 | 1.2068 |
| 296 | SABS | 2018 | September | 0.0343 | 1.1363 |
| 297 | SABS | 2018 | September | 0.0281 | 1.1358 |
| 298 | SABS | 2018 | September | 0.0262 | 1.1476 |
| 299 | SABS | 2018 | September | 0.0275 | 1.1398 |
| 300 | SABS | 2018 | September | 0.0285 | 1.1318 |
| 301 | SABS | 2018 | September | 0.0265 | 1.1766 |
| 302 | SABS | 2018 | September | 0.0245 | 1.1206 |
| 303 | SABS | 2018 | September | 0.0243 | 1.5636 |
| 304 | SABS | 2018 | September | 0.0088 | 1.5624 |
| 305 | SABS | 2018 | September | 0.0062 | 1.5642 |
| 306 | SABS | 2018 | September | 0.0134 | 1.5342 |
| 307 | SABS | 2018 | September | 0.0054 | 1.5560 |
| 308 | SABS | 2018 | September | 0.0066 | 1.5772 |
| 309 | SABS | 2018 | September | 0.0052 | 1.5885 |
| 310 | SABS | 2018 | September | 0.0052 | 1.6083 |
| 311 | SABS | 2018 | September | 0.0158 | 1.5192 |
| 312 | SABS | 2018 | September | 0.0250 | 1.5253 |
| 313 | SABS | 2018 | September | 0.1761 | 1.5811 |
| 314 | SABS | 2018 | September | 0.2509 | 1.8724 |
| 315 | SABS | 2018 | September | 0.2509 | 1.8726 |
| 316 | SABS | 2018 | September | 0.1067 | 1.8448 |
| 317 | SABS | 2018 | September | 0.2811 | 1.7275 |
| 318 | SABS | 2018 | September | 0.3092 | 1.7272 |
| 319 | SABS | 2018 | September | 0.3092 | 1.7271 |
| 320 | SABS | 2018 | September | 0.2007 | 1.5986 |
| 321 | SABS | 2018 | September | 0.2007 | 1.5986 |
| 322 | SABS | 2018 | September | 0.2323 | 1.5417 |
| 323 | SABS | 2018 | September | 0.1091 | 1.3596 |
| 324 | SABS | 2018 | September | 0.0264 | 1.4286 |
| 325 | SABS | 2018 | September | 0.0503 | 1.5388 |
| 326 | SABS | 2018 | September | 0.0584 | 1.5224 |
| 327 | SABS | 2018 | September | 0.0475 | 1.5374 |
| 328 | SABS | 2018 | September | 0.0310 | 1.4894 |
| 329 | SABS | 2018 | September | 0.0585 | 1.5224 |
| 330 | SABS | 2018 | September | 0.0178 | 1.5409 |
| 331 | SABS | 2018 | September | 0.0586 | 1.4706 |
| 332 | SABS | 2018 | September | 0.0103 | 1.5474 |
| 333 | SABS | 2018 | September | 0.4288 | 1.5125 |
| 334 | SABS | 2018 | September | 0.5712 | 1.4879 |
| 335 | SABS | 2018 | September | 0.5380 | 1.4575 |
| 336 | SABS | 2018 | September | 0.5666 | 1.4680 |
| 337 | SABS | 2018 | September | 0.5339 | 1.4198 |
| 338 | SABS | 2018 | September | 0.5572 | 1.5390 |
| 339 | SABS | 2018 | September | 0.0414 | 1.3932 |
| 340 | SABS | 2018 | September | 0.2299 | 1.4614 |
| 341 | SABS | 2018 | September | 0.0679 | 1.2977 |
| 342 | SABS | 2018 | September | 0.0096 | 1.5008 |
| 343 | SABS | 2018 | September | 0.1208 | 1.2947 |
| 344 | SABS | 2018 | September | 0.1915 | 1.2833 |
| 345 | SABS | 2018 | September | 0.0687 | 1.2363 |
| 346 | SABS | 2018 | September | 0.1032 | 1.2242 |
| 347 | SABS | 2018 | September | 0.0560 | 1.3697 |
| 348 | SABS | 2018 | September | 0.0990 | 1.2613 |
| 349 | SABS | 2018 | September | 0.0462 | 1.2443 |
| 350 | SABS | 2018 | September | 0.1435 | 1.2123 |
| 351 | SABS | 2018 | September | 0.0948 | 1.2664 |
| 352 | SABS | 2018 | September | 0.0691 | 1.2246 |
| 353 | SABS | 2018 | September | 0.5086 | 1.5185 |
| 354 | SABS | 2018 | September | 0.1872 | 1.5158 |
| 355 | SABS | 2018 | September | 0.2603 | 1.4654 |
| 356 | SABS | 2018 | September | 0.1756 | 1.6366 |
| 357 | SABS | 2018 | September | 0.2032 | 1.5310 |
| 358 | SABS | 2018 | September | 0.1301 | 1.5616 |
| 359 | SABS | 2018 | September | 0.1006 | 1.6423 |
| 360 | SABS | 2018 | September | 0.0817 | 1.5433 |
| 361 | SABS | 2018 | September | 0.2580 | 1.4700 |
| 362 | SABS | 2018 | September | 0.4709 | 1.5062 |
| 363 | SABS | 2019 | September | 0.0385 | 1.9488 |
| 364 | SABS | 2019 | September | 0.0411 | 2.1881 |
| 365 | SABS | 2019 | September | 0.0298 | 1.9445 |
| 366 | SABS | 2019 | September | 0.0693 | 2.1120 |
| 367 | SABS | 2019 | September | 0.0826 | 2.0553 |
| 368 | SABS | 2019 | September | 0.0514 | 1.9695 |
| 369 | SABS | 2019 | September | 0.0697 | 1.9594 |
| 370 | SABS | 2019 | September | 0.0490 | 1.9290 |
| 371 | SABS | 2019 | September | 0.0401 | 1.8786 |
| 372 | SABS | 2019 | September | 0.0413 | 1.8814 |
| 373 | SABS | 2019 | September | 0.0833 | 2.2541 |
| 374 | SABS | 2019 | September | 0.2411 | 2.3915 |
| 375 | SABS | 2019 | September | 0.1986 | 2.3093 |
| 376 | SABS | 2019 | September | 0.0702 | 2.2457 |
| 377 | SABS | 2019 | September | 0.1079 | 2.2041 |
| 378 | SABS | 2019 | September | 0.0871 | 2.2450 |
| 379 | SABS | 2019 | September | 0.0342 | 1.8264 |
| 380 | SABS | 2019 | September | 0.0920 | 2.2411 |
| 381 | SABS | 2019 | September | 0.0994 | 2.1965 |
| 382 | SABS | 2019 | September | 0.0867 | 2.2238 |
| 383 | SABS | 2019 | September | 0.0255 | 1.6944 |
| 384 | SABS | 2019 | September | 0.0256 | 1.6527 |
| 385 | SABS | 2019 | September | 0.0252 | 1.6444 |
| 386 | SABS | 2019 | September | 0.0270 | 1.6413 |
| 387 | SABS | 2019 | September | 0.0270 | 1.6671 |
| 388 | SABS | 2019 | September | 0.0275 | 1.6474 |
| 389 | SABS | 2019 | September | 0.0279 | 1.7299 |
| 390 | SABS | 2019 | September | 0.0278 | 1.7940 |
| 391 | SABS | 2019 | September | 0.0689 | 2.0384 |
| 392 | SABS | 2019 | September | 0.0277 | 1.8433 |
| 393 | SABS | 2019 | September | 0.0304 | 1.9027 |
| 394 | SABS | 2019 | September | 0.0262 | 1.7854 |
| 395 | SABS | 2019 | September | 0.0259 | 1.7261 |
| 396 | SABS | 2019 | September | 0.0268 | 1.7430 |
| 397 | SABS | 2019 | September | 0.0313 | 1.7781 |
| 398 | SABS | 2019 | September | 0.0302 | 1.8764 |
| 399 | SABS | 2019 | September | 0.0289 | 1.9491 |
| 400 | SABS | 2019 | September | 0.0353 | 1.9686 |
| 401 | SABS | 2019 | September | 0.0964 | 2.0812 |
| 402 | SABS | 2019 | September | 0.0334 | 1.8143 |
| 403 | SABS | 2019 | September | 0.0276 | 1.8576 |
| 404 | SABS | 2019 | September | 0.0263 | 1.8484 |
| 405 | SABS | 2019 | September | 0.0257 | 1.8379 |
| 406 | SABS | 2019 | September | 0.0267 | 1.8945 |
| 407 | SABS | 2019 | September | 0.0257 | 1.8890 |
| 408 | SABS | 2019 | September | 0.0275 | 1.8974 |
| 409 | SABS | 2019 | September | 0.0411 | 1.9034 |
| 410 | SABS | 2019 | September | 0.0288 | 1.9136 |
| 411 | SABS | 2019 | September | 0.0797 | 2.0030 |
| 412 | SABS | 2019 | September | 0.0336 | 1.8649 |
| 413 | SABS | 2019 | September | 0.0295 | 1.9122 |
| 414 | SABS | 2019 | September | 0.0270 | 1.8633 |
| 415 | SABS | 2019 | September | 0.0288 | 1.9240 |
| 416 | SABS | 2019 | September | 0.0279 | 1.8692 |
| 417 | SABS | 2019 | September | 0.0269 | 1.8349 |
| 418 | SABS | 2019 | September | 0.0267 | 1.8900 |
| 419 | SABS | 2019 | September | 0.0266 | 1.8987 |
| 420 | SABS | 2019 | September | 0.0289 | 1.9509 |
| 421 | SABS | 2019 | September | 0.1130 | 2.1296 |
| 422 | SABS | 2019 | September | 0.0408 | 1.9244 |
| 423 | SABS | 2019 | September | 0.0276 | 1.8958 |
| 424 | SABS | 2019 | September | 0.0270 | 1.7825 |
| 425 | SABS | 2019 | September | 0.0246 | 1.8143 |
| 426 | SABS | 2019 | September | 0.0254 | 1.8340 |
| 427 | SABS | 2019 | September | 0.0264 | 1.7939 |
| 428 | SABS | 2019 | September | 0.0253 | 1.7497 |
| 429 | SABS | 2019 | September | 0.0254 | 1.7524 |
| 430 | SABS | 2019 | September | 0.0289 | 1.7851 |
| 431 | SABS | 2019 | September | 0.1022 | 2.0679 |
| 432 | SABS | 2019 | September | 0.0977 | 1.9913 |
| 433 | SABS | 2019 | September | 0.0582 | 1.8833 |
| 434 | SABS | 2019 | September | 0.0446 | 1.8481 |
| 435 | SABS | 2019 | September | 0.0433 | 1.8605 |
| 436 | SABS | 2019 | September | 0.0316 | 1.8258 |
| 437 | SABS | 2019 | September | 0.0297 | 1.8472 |
| 438 | SABS | 2019 | September | 0.0269 | 1.8242 |
| 439 | SABS | 2019 | September | 0.0274 | 1.8261 |
| 440 | SABS | 2019 | September | 0.0287 | 1.8121 |
| 441 | SABS | 2019 | September | 0.1133 | 2.0848 |
| 442 | SABS | 2019 | September | 0.0766 | 1.9680 |
| 443 | SABS | 2019 | September | 0.0433 | 1.9490 |
| 444 | SABS | 2019 | September | 0.1876 | 2.2198 |
| 445 | SABS | 2019 | September | 0.2378 | 2.2619 |
| 446 | SABS | 2019 | September | 0.1375 | 2.2064 |
| 447 | SABS | 2019 | September | 0.0566 | 1.9139 |
| 448 | SABS | 2019 | September | 0.1336 | 2.2096 |
| 449 | SABS | 2019 | September | 0.0905 | 2.0937 |
| 450 | SABS | 2019 | September | 0.0948 | 2.0651 |
| 451 | SABS | 2019 | September | 0.1037 | 2.2538 |
| 452 | SABS | 2019 | September | 0.0407 | 1.7897 |
| 453 | SABS | 2019 | September | 0.0390 | 1.9047 |
| 454 | SABS | 2019 | September | 0.0367 | 1.7407 |
| 455 | SABS | 2019 | September | 0.0317 | 1.9143 |
| 456 | SABS | 2019 | September | 0.0421 | 2.0754 |
| 457 | SABS | 2019 | September | 0.0403 | 1.9244 |
| 458 | SABS | 2019 | September | 0.0786 | 2.0144 |
| 459 | SABS | 2019 | September | 0.0543 | 1.9363 |
| 460 | SABS | 2019 | September | 0.0534 | 2.0608 |
| 461 | SABS | 2019 | September | 0.0965 | 2.2814 |
| 462 | SABS | 2019 | September | 0.0426 | 1.9618 |
| 463 | SABS | 2019 | September | 0.0329 | 1.8720 |
| 464 | SABS | 2019 | September | 0.0352 | 2.1287 |
| 465 | SABS | 2019 | September | 0.0486 | 2.0467 |
| 466 | SABS | 2019 | September | 0.0322 | 2.1289 |
| 467 | SABS | 2019 | September | 0.0481 | 1.8534 |
| 468 | SABS | 2019 | September | 0.0330 | 2.0984 |
| 469 | SABS | 2019 | September | 0.0462 | 1.9796 |
| 470 | SABS | 2019 | September | 0.0584 | 2.0854 |
| 471 | SABS | 2018 | December | 0.8687 | 1.2542 |
| 472 | SABS | 2018 | December | 0.9941 | 0.2489 |
| 473 | SABS | 2018 | December | 0.9032 | 0.7791 |
| 474 | SABS | 2018 | December | 0.7076 | 1.4236 |
| 475 | SABS | 2018 | December | 0.4491 | 1.5079 |
| 476 | SABS | 2018 | December | 0.3309 | 1.3944 |
| 477 | SABS | 2018 | December | 0.7270 | 1.1723 |
| 478 | SABS | 2018 | December | 0.8986 | 0.7198 |
| 479 | SABS | 2018 | December | 0.8835 | 0.9351 |
| 480 | SABS | 2018 | December | 0.7424 | 1.1411 |
| 481 | SABS | 2018 | December | 0.5523 | 1.5778 |
| 482 | SABS | 2018 | December | 0.8609 | 1.2840 |
| 483 | SABS | 2018 | December | 0.7813 | 1.3772 |
| 484 | SABS | 2018 | December | 0.7172 | 1.5897 |
| 485 | SABS | 2018 | December | 0.8664 | 1.1483 |
| 486 | SABS | 2018 | December | 0.9209 | 0.9598 |
| 487 | SABS | 2018 | December | 0.8506 | 1.4005 |
| 488 | SABS | 2018 | December | 0.8863 | 1.1959 |
| 489 | SABS | 2018 | December | 0.8664 | 1.1945 |
| 490 | SABS | 2018 | December | 0.9455 | 0.8197 |
| 491 | SABS | 2018 | December | 0.9135 | 1.1269 |
| 492 | SABS | 2018 | December | 0.9035 | 1.1155 |
| 493 | SABS | 2018 | December | 0.8778 | 1.2352 |
| 494 | SABS | 2018 | December | 0.8736 | 1.1137 |
| 495 | SABS | 2018 | December | 0.6010 | 1.9363 |
| 496 | SABS | 2018 | December | 0.3983 | 2.0373 |
| 497 | SABS | 2018 | December | 0.6621 | 1.9525 |
| 498 | SABS | 2018 | December | 0.6408 | 2.0080 |
| 499 | SABS | 2018 | December | 0.8878 | 0.7442 |
| 500 | SABS | 2018 | December | 0.8705 | 0.9583 |
| 501 | SABS | 2018 | December | 0.7987 | 1.6976 |
| 502 | SABS | 2018 | December | 0.8160 | 1.6367 |
| 503 | SABS | 2018 | December | 0.7002 | 2.0581 |
| 504 | SABS | 2018 | December | 0.7091 | 2.0485 |
| 505 | SABS | 2018 | December | 0.7320 | 1.9771 |
| 506 | SABS | 2018 | December | 0.6706 | 2.1874 |
| 507 | SABS | 2018 | December | 0.6858 | 2.1390 |
| 508 | SABS | 2018 | December | 0.7387 | 1.9496 |
| 509 | SABS | 2018 | December | 0.6712 | 2.0479 |
| 510 | SABS | 2018 | December | 0.7096 | 2.0628 |
| 511 | SABS | 2018 | December | 0.6862 | 1.9964 |
| 512 | SABS | 2018 | December | 0.6759 | 2.0311 |
| 513 | SABS | 2018 | December | 0.6752 | 1.9973 |
| 514 | SABS | 2018 | December | 0.6980 | 2.0136 |
| 515 | SABS | 2018 | December | 0.7170 | 2.0230 |
| 516 | SABS | 2018 | December | 0.7725 | 1.8273 |
| 517 | SABS | 2018 | December | 0.7734 | 1.8040 |
| 518 | SABS | 2018 | December | 0.7572 | 1.8656 |
| 519 | SABS | 2018 | December | 0.7000 | 2.0515 |
| 520 | SABS | 2018 | December | 0.7005 | 2.0668 |
| 521 | SABS | 2018 | December | 0.6754 | 2.0901 |
| 522 | SABS | 2018 | December | 0.7052 | 2.0441 |
| 523 | SABS | 2018 | December | 0.7003 | 2.0905 |
| 524 | SABS | 2018 | December | 0.6892 | 2.1161 |
| 525 | SABS | 2018 | December | 0.6485 | 2.1233 |
| 526 | SABS | 2018 | December | 0.6815 | 2.0817 |
| 527 | SABS | 2018 | December | 0.7054 | 2.0699 |
| 528 | SABS | 2018 | December | 0.7042 | 2.0802 |
| 529 | SABS | 2018 | December | 0.7350 | 1.9803 |
| 530 | SABS | 2018 | December | 0.7377 | 1.9357 |
| 531 | SABS | 2018 | December | 0.6942 | 2.0268 |
| 532 | SABS | 2018 | December | 0.6990 | 2.0789 |
| 533 | SABS | 2018 | December | 0.7152 | 2.0335 |
| 534 | SABS | 2018 | December | 0.7463 | 1.9306 |
| 535 | SABS | 2018 | December | 0.7409 | 1.9421 |
| 536 | SABS | 2018 | December | 0.7179 | 2.0228 |
| 537 | SABS | 2018 | December | 0.7091 | 2.0579 |
| 538 | SABS | 2018 | December | 0.6908 | 2.0347 |
| 539 | SABS | 2018 | December | 0.6484 | 1.8860 |
| 540 | SABS | 2018 | December | 0.6902 | 2.0073 |
| 541 | SABS | 2018 | December | 0.7231 | 1.9716 |
| 542 | SABS | 2018 | December | 0.7087 | 1.9763 |
| 543 | SABS | 2018 | December | 0.7283 | 1.9773 |
| 544 | SABS | 2018 | December | 0.5782 | 2.1959 |
| 545 | SABS | 2018 | December | 0.6463 | 2.2106 |
| 546 | SABS | 2018 | December | 0.7182 | 2.0284 |
| 547 | SABS | 2018 | December | 0.7234 | 2.0127 |
| 548 | SABS | 2018 | December | 0.7243 | 2.0032 |
| 549 | SABS | 2018 | December | 0.6918 | 2.1154 |
| 550 | SABS | 2018 | December | 0.6758 | 2.1171 |
| 551 | SABS | 2018 | December | 0.9994 | 0.0828 |
| 552 | SABS | 2018 | December | 0.7146 | 2.0112 |
| 553 | SABS | 2018 | December | 0.6927 | 2.0677 |
| 554 | SABS | 2018 | December | 0.7105 | 2.0469 |
| 555 | SABS | 2018 | December | 0.7379 | 1.9494 |
| 556 | SABS | 2018 | December | 0.6804 | 2.0754 |
| 557 | SABS | 2018 | December | 0.7448 | 1.8880 |
| 558 | SABS | 2018 | December | 0.7327 | 1.8974 |
| 559 | SABS | 2018 | December | 0.7505 | 1.9079 |
| 560 | SABS | 2018 | December | 0.9213 | 1.0600 |
| 561 | SABS | 2018 | December | 0.7075 | 1.9889 |
| 562 | SABS | 2018 | December | 0.7428 | 1.9049 |
| 563 | SABS | 2018 | December | 0.7262 | 1.9981 |
| 564 | SABS | 2018 | December | 0.6937 | 2.0310 |
| 565 | SABS | 2018 | December | 0.5930 | 2.0560 |
| 566 | SABS | 2018 | December | 0.7074 | 1.7838 |
| 567 | SABS | 2018 | December | 0.6957 | 1.9236 |
| 568 | SABS | 2018 | December | 0.7042 | 1.9273 |
| 569 | SABS | 2018 | December | 0.8372 | 1.6071 |
| 570 | SABS | 2018 | December | 0.8119 | 1.6634 |
| 571 | SABS | 2018 | December | 0.8546 | 1.5119 |
| 572 | SABS | 2018 | December | 0.8061 | 1.7359 |
| 573 | SABS | 2018 | December | 0.8516 | 1.5156 |
| 574 | SABS | 2018 | December | 0.8855 | 1.3551 |
| 575 | SABS | 2018 | December | 0.7405 | 1.9542 |
| 576 | SABS | 2018 | December | 0.7256 | 1.9725 |
| 577 | SABS | 2018 | December | 0.6988 | 2.0650 |
| 578 | SABS | 2018 | December | 0.6832 | 2.0941 |
| 579 | SABS | 2018 | December | 0.7158 | 2.0348 |
| 580 | SABS | 2018 | December | 0.7718 | 1.8343 |
| 581 | SABS | 2018 | December | 0.7273 | 1.9606 |
| 582 | SABS | 2018 | December | 0.6883 | 2.1061 |
| 583 | SABS | 2018 | December | 0.4751 | 2.0317 |
| 584 | SABS | 2018 | December | 0.6854 | 2.1210 |
| 585 | SABS | 2018 | December | 0.6863 | 2.0969 |
| 586 | SABS | 2018 | December | 0.8198 | 1.6661 |
| 587 | SABS | 2018 | December | 0.8167 | 1.6784 |
| 588 | SABS | 2018 | December | 0.6040 | 2.2452 |
| 589 | SABS | 2018 | December | 0.6131 | 2.2943 |
| 590 | SABS | 2018 | December | 0.6187 | 2.2085 |
| 591 | SABS | 2018 | December | 0.7689 | 1.8416 |
| 592 | SABS | 2018 | December | 0.5815 | 2.3224 |
| 593 | SABS | 2018 | December | 0.5945 | 2.2776 |
| 594 | SABS | 2018 | December | 0.5447 | 2.4035 |
| 595 | SABS | 2018 | December | 0.5728 | 2.3132 |
| 596 | SABS | 2018 | December | 0.2991 | 2.0412 |
| 597 | SABS | 2018 | December | 0.4831 | 2.2696 |
| 598 | SABS | 2019 | December | 0.9151 | 0.8279 |
| 599 | SABS | 2019 | December | 0.7869 | 1.3262 |
| 600 | SABS | 2019 | December | 0.9149 | 0.8687 |
| 601 | SABS | 2019 | December | 0.8544 | 1.1628 |
| 602 | SABS | 2019 | December | 0.9532 | 0.6522 |
| 603 | SABS | 2019 | December | 0.9848 | 0.3853 |
| 604 | SABS | 2019 | December | 0.9809 | 0.4263 |
| 605 | SABS | 2019 | December | 0.9843 | 0.3895 |
| 606 | SABS | 2019 | December | 0.9739 | 0.4848 |
| 607 | SABS | 2019 | December | 0.9383 | 0.6894 |
| 608 | SABS | 2019 | December | 0.8560 | 1.1873 |
| 609 | SABS | 2019 | December | 0.6394 | 1.8290 |
| 610 | SABS | 2019 | December | 0.6604 | 1.7725 |
| 611 | SABS | 2019 | December | 0.6608 | 1.8239 |
| 612 | SABS | 2019 | December | 0.3879 | 2.1807 |
| 613 | SABS | 2019 | December | 0.6484 | 1.7589 |
| 614 | SABS | 2019 | December | 0.6897 | 1.7163 |
| 615 | SABS | 2019 | December | 0.4087 | 2.0446 |
| 616 | SABS | 2019 | December | 0.9232 | 0.8871 |
| 617 | SABS | 2019 | December | 0.3017 | 1.6675 |
| 618 | SABS | 2019 | December | 0.9993 | 0.0909 |
| 619 | SABS | 2019 | December | 0.9997 | 0.0553 |
| 620 | SABS | 2019 | December | 0.9994 | 0.0801 |
| 621 | SABS | 2019 | December | 0.9997 | 0.0614 |
| 622 | SABS | 2019 | December | 0.9998 | 0.0518 |
| 623 | SABS | 2019 | December | 0.9999 | 0.0323 |
| 624 | SABS | 2019 | December | 0.9986 | 0.1026 |
| 625 | SABS | 2019 | December | 0.9989 | 0.0881 |
| 626 | SABS | 2019 | December | 0.7276 | 1.8595 |
| 627 | SABS | 2019 | December | 0.7673 | 1.6458 |
| 628 | SABS | 2019 | December | 0.5099 | 2.1986 |
| 629 | SABS | 2019 | December | 0.4575 | 2.2243 |
| 630 | SABS | 2019 | December | 0.4183 | 2.2191 |
| 631 | SABS | 2019 | December | 0.2517 | 2.2149 |
| 632 | SABS | 2019 | December | 0.3565 | 2.3070 |
| 633 | SABS | 2019 | December | 0.2553 | 2.2454 |
| 634 | SABS | 2019 | December | 0.4586 | 2.1005 |
| 635 | SABS | 2019 | December | 0.1217 | 2.1991 |
| 636 | SABS | 2019 | December | 0.3190 | 2.4986 |
| 637 | SABS | 2019 | December | 0.1744 | 2.5036 |
| 638 | SABS | 2019 | December | 0.3037 | 2.6599 |
| 639 | SABS | 2019 | December | 0.2644 | 2.5756 |
| 640 | SABS | 2019 | December | 0.4422 | 2.4680 |
| 641 | SABS | 2019 | December | 0.3307 | 2.5426 |
| 642 | SABS | 2019 | December | 0.2146 | 2.1953 |
| 643 | SABS | 2019 | December | 0.1066 | 1.8370 |
| 644 | SABS | 2019 | December | 0.4943 | 2.4948 |
| 645 | SABS | 2019 | December | 0.5336 | 2.4649 |
| 646 | SABS | 2019 | December | 0.5909 | 2.3944 |
| 647 | SABS | 2019 | December | 0.4587 | 2.4530 |
| 648 | SABS | 2019 | December | 0.5523 | 2.4636 |
| 649 | SABS | 2019 | December | 0.5745 | 2.4249 |
| 650 | SABS | 2019 | December | 0.6158 | 2.3401 |
| 651 | SABS | 2019 | December | 0.8610 | 1.4562 |
| 652 | SABS | 2019 | December | 0.8999 | 1.2808 |
| 653 | SABS | 2019 | December | 0.9092 | 1.2163 |
| 654 | SABS | 2019 | December | 0.8470 | 1.5681 |
| 655 | SABS | 2019 | December | 0.8807 | 1.3768 |
| 656 | SABS | 2019 | December | 0.7105 | 1.5682 |
| 657 | SABS | 2019 | December | 0.2782 | 2.0538 |
| 658 | SABS | 2019 | December | 0.3020 | 1.7387 |
| 659 | SABS | 2019 | December | 0.3504 | 1.7803 |
| 660 | SABS | 2019 | December | 0.6124 | 2.0437 |
| 661 | SABS | 2019 | December | 0.5215 | 1.5803 |
| 662 | SABS | 2019 | December | 0.6431 | 1.9031 |
| 663 | SABS | 2019 | December | 0.4565 | 1.9208 |
| 664 | SABS | 2019 | December | 0.2823 | 1.6640 |
| 665 | SABS | 2019 | December | 0.6176 | 2.2173 |
| 666 | SABS | 2019 | December | 0.9729 | 0.7825 |
| 667 | SABS | 2019 | December | 0.5309 | 0.8964 |
| 668 | SABS | 2019 | December | 0.8468 | 1.5748 |
| 669 | SABS | 2019 | December | 0.9747 | 0.5433 |
| 670 | SABS | 2019 | December | 0.9230 | 0.8484 |
| 671 | SABS | 2019 | December | 0.6024 | 0.8263 |
| 672 | SABS | 2019 | December | 0.9871 | 0.4438 |
| 673 | SABS | 2019 | December | 0.5939 | 0.8348 |
| 674 | SABS | 2019 | December | 0.1349 | 1.0117 |
| 675 | SABS | 2019 | December | 0.9145 | 1.1738 |
| 676 | SABS | 2019 | December | 0.9989 | 0.1265 |
| 677 | SABS | 2019 | December | 0.6121 | 2.2750 |
| 678 | SABS | 2019 | December | 0.5220 | 2.1054 |
| 679 | SABS | 2019 | December | 0.9952 | 0.2575 |
| 680 | SABS | 2019 | December | 0.3155 | 2.2083 |
| 681 | SABS | 2019 | December | 0.6455 | 2.2612 |
| 682 | SABS | 2019 | December | 0.8318 | 1.6348 |
| 683 | SABS | 2019 | December | 0.9997 | 0.0667 |
| 684 | SABS | 2019 | December | 0.8842 | 1.3408 |
| 685 | SABS | 2019 | December | 0.9359 | 1.0169 |
| 686 | SABS | 2019 | December | 0.5586 | 2.4642 |
| 687 | SABS | 2019 | December | 0.6149 | 2.3483 |
| 688 | SABS | 2019 | December | 0.5498 | 2.4878 |
| 689 | SABS | 2019 | December | 0.5481 | 2.4788 |
| 690 | SABS | 2019 | December | 0.5270 | 2.5232 |
| 691 | SABS | 2019 | December | 0.1652 | 1.8128 |
| 692 | SABS | 2019 | December | 0.4955 | 2.5701 |
| 693 | SABS | 2019 | December | 0.0214 | 1.2962 |
| 694 | SABS | 2019 | December | 0.5741 | 2.4194 |
| 695 | SABS | 2019 | December | 0.1232 | 1.6346 |
| 696 | SABS | 2019 | December | 0.8979 | 0.9641 |
| 697 | SABS | 2019 | December | 0.9984 | 0.5580 |
| 698 | SABS | 2019 | December | 0.8862 | 0.9364 |
| 699 | SABS | 2019 | December | 0.9981 | 0.1583 |
| 700 | SABS | 2019 | December | 0.9908 | 0.3203 |
| 701 | SABS | 2019 | December | 0.9911 | 0.3738 |
| 702 | SABS | 2019 | December | 0.5413 | 2.0115 |
| 703 | SABS | 2019 | December | 0.4979 | 2.4968 |
| 704 | SABS | 2019 | December | 0.1843 | 1.5320 |
| 705 | SABS | 2019 | December | 0.1263 | 1.7111 |
| 706 | SABS | 2019 | December | 0.8136 | 1.4696 |
| 707 | SABS | 2019 | December | 0.8211 | 1.3512 |
| 708 | SABS | 2019 | December | 0.8217 | 1.5760 |
| 709 | SABS | 2019 | December | 0.5933 | 2.2360 |
| 710 | SABS | 2019 | December | 0.2471 | 2.2301 |
| 711 | SABS | 2019 | December | 0.2030 | 2.0891 |
| 712 | SABS | 2019 | December | 0.4725 | 2.4304 |
| 713 | SABS | 2019 | December | 0.2516 | 2.4003 |
| 714 | SABS | 2019 | December | 0.1503 | 2.3704 |
| 715 | SABS | 2019 | December | 0.1508 | 2.0131 |
| 716 | SABS | 2019 | December | 0.1050 | 1.8771 |
| 717 | SABS | 2019 | December | 0.1025 | 1.3503 |
| 718 | SABS | 2019 | December | 0.1606 | 1.4523 |
| 719 | SABS | 2019 | December | 0.2345 | 1.4185 |
| 720 | SABS | 2019 | December | 0.4224 | 1.4523 |
| 721 | SABS | 2019 | December | 0.2569 | 1.4779 |
| 722 | SABS | 2019 | December | 0.1088 | 1.6132 |
| 723 | SABS | 2019 | December | 0.6877 | 1.2676 |
| 724 | IK | 2018 | April | 0.0036 | 1.5592 |
| 725 | IK | 2018 | April | 0.0082 | 1.4881 |
| 726 | IK | 2018 | April | 0.0098 | 1.5280 |
| 727 | IK | 2018 | April | 0.0035 | 1.4197 |
| 728 | IK | 2018 | April | 0.0019 | 1.4474 |
| 729 | IK | 2018 | April | 0.0037 | 1.4335 |
| 730 | IK | 2018 | April | 0.0014 | 1.4234 |
| 731 | IK | 2018 | April | 0.0020 | 1.3417 |
| 732 | IK | 2018 | April | 0.0015 | 1.3336 |
| 733 | IK | 2018 | April | 0.0074 | 1.4606 |
| 734 | IK | 2018 | April | 0.0025 | 1.2471 |
| 735 | IK | 2018 | April | 0.0040 | 1.2550 |
| 736 | IK | 2018 | April | 0.0068 | 1.2730 |
| 737 | IK | 2018 | April | 0.0063 | 1.3212 |
| 738 | IK | 2018 | April | 0.0055 | 1.3071 |
| 739 | IK | 2018 | April | 0.0564 | 1.2732 |
| 740 | IK | 2018 | April | 0.4616 | 2.1873 |
| 741 | IK | 2018 | April | 0.3689 | 2.0419 |
| 742 | IK | 2018 | April | 0.4294 | 1.9674 |
| 743 | IK | 2018 | April | 0.7272 | 1.6635 |
| 744 | IK | 2018 | April | 0.5706 | 1.7906 |
| 745 | IK | 2018 | April | 0.0716 | 1.2929 |
| 746 | IK | 2018 | April | 0.1266 | 1.3017 |
| 747 | IK | 2018 | April | 0.0112 | 1.4165 |
| 748 | IK | 2018 | April | 0.1635 | 1.5304 |
| 749 | IK | 2018 | April | 0.9570 | 0.6118 |
| 750 | IK | 2018 | April | 0.9975 | 0.1646 |
| 751 | IK | 2018 | April | 0.8854 | 1.1186 |
| 752 | IK | 2018 | April | 0.7449 | 1.6046 |
| 753 | IK | 2018 | April | 0.8322 | 1.2231 |
| 754 | IK | 2018 | April | 0.6181 | 1.0651 |
| 755 | IK | 2018 | April | 0.7156 | 1.5534 |
| 756 | IK | 2018 | April | 0.7395 | 1.4026 |
| 757 | IK | 2018 | April | 0.0347 | 1.4861 |
| 758 | IK | 2018 | April | 0.8602 | 1.1715 |
| 759 | IK | 2018 | April | 0.8249 | 1.1539 |
| 760 | IK | 2018 | April | 0.8535 | 0.8536 |
| 761 | IK | 2018 | April | 0.8325 | 0.7324 |
| 762 | IK | 2018 | April | 0.8027 | 0.8243 |
| 763 | IK | 2018 | April | 0.8001 | 0.7906 |
| 764 | IK | 2018 | April | 0.6398 | 1.1578 |
| 765 | IK | 2018 | April | 0.8042 | 0.8837 |
| 766 | IK | 2018 | April | 0.9638 | 0.4310 |
| 767 | IK | 2018 | April | 0.9208 | 0.6008 |
| 768 | IK | 2018 | April | 0.5755 | 1.6191 |
| 769 | IK | 2018 | April | 0.9589 | 0.6085 |
| 770 | IK | 2018 | April | 0.7749 | 1.3277 |
| 771 | IK | 2018 | April | 0.6481 | 1.6156 |
| 772 | IK | 2018 | April | 0.6841 | 1.6681 |
| 773 | IK | 2018 | April | 0.8634 | 0.6744 |
| 774 | IK | 2018 | April | 0.9990 | 0.0749 |
| 775 | IK | 2018 | April | 0.9674 | 0.4237 |
| 776 | IK | 2018 | April | 0.9672 | 0.3859 |
| 777 | IK | 2018 | April | 0.3980 | 1.2195 |
| 778 | IK | 2018 | April | 0.9626 | 0.4441 |
| 779 | IK | 2018 | April | 0.9873 | 0.2871 |
| 780 | IK | 2018 | April | 0.7293 | 1.2265 |
| 781 | IK | 2018 | April | 0.5366 | 1.4059 |
| 782 | IK | 2018 | April | 0.5786 | 1.4294 |
| 783 | IK | 2018 | April | 0.7210 | 1.0944 |
| 784 | IK | 2018 | April | 0.0901 | 0.1023 |
| 785 | IK | 2018 | April | 0.4969 | 1.5068 |
| 786 | IK | 2018 | April | 0.4481 | 1.0255 |
| 787 | IK | 2018 | April | 0.4497 | 1.6986 |
| 788 | IK | 2018 | April | 0.4898 | 1.0547 |
| 789 | IK | 2018 | April | 0.4924 | 1.0442 |
| 790 | IK | 2018 | April | 0.4479 | 1.1705 |
| 791 | IK | 2018 | April | 0.3447 | 0.5422 |
| 792 | IK | 2018 | April | 0.4213 | 0.8248 |
| 793 | IK | 2018 | April | 0.4000 | 0.9431 |
| 794 | IK | 2018 | April | 0.6207 | 1.7729 |
| 795 | IK | 2018 | April | 0.8206 | 1.2299 |
| 796 | IK | 2018 | April | 0.1192 | 2.1139 |
| 797 | IK | 2018 | April | 0.3424 | 1.9185 |
| 798 | IK | 2018 | April | 0.2381 | 1.9382 |
| 799 | IK | 2018 | April | 0.4851 | 1.9118 |
| 800 | IK | 2018 | April | 0.3378 | 1.9930 |
| 801 | IK | 2018 | April | 0.2263 | 1.9481 |
| 802 | IK | 2018 | April | 0.4986 | 1.8185 |
| 803 | IK | 2018 | April | 0.1990 | 1.9686 |
| 804 | IK | 2018 | April | 0.3587 | 1.1164 |
| 805 | IK | 2018 | April | 0.5817 | 1.6747 |
| 806 | IK | 2018 | April | 0.9972 | 0.1668 |
| 807 | IK | 2018 | April | 0.9945 | 0.2583 |
| 808 | IK | 2018 | April | 0.8899 | 1.0705 |
| 809 | IK | 2018 | April | 0.9307 | 0.8344 |
| 810 | IK | 2018 | April | 0.9850 | 0.3812 |
| 811 | IK | 2018 | April | 0.9994 | 0.0747 |
| 812 | IK | 2018 | April | 0.9997 | 0.0534 |
| 813 | IK | 2018 | April | 0.9965 | 0.1316 |
| 814 | IK | 2018 | April | 0.3127 | 1.4888 |
| 815 | IK | 2018 | April | 0.3709 | 1.3790 |
| 816 | IK | 2018 | April | 0.3994 | 1.2301 |
| 817 | IK | 2018 | April | 0.0846 | 1.3603 |
| 818 | IK | 2018 | April | 0.0761 | 1.3447 |
| 819 | IK | 2018 | April | 0.0261 | 1.2736 |
| 820 | IK | 2018 | April | 0.0008 | 1.0534 |
| 821 | IK | 2018 | April | 0.0009 | 1.0674 |
| 822 | IK | 2018 | April | 0.0146 | 1.4951 |
| 823 | IK | 2018 | April | 0.0008 | 1.0776 |
| 824 | IK | 2018 | April | 0.0648 | 1.3888 |
| 825 | IK | 2018 | April | 0.1720 | 1.7849 |
| 826 | IK | 2018 | April | 0.0041 | 1.2114 |
| 827 | IK | 2018 | April | 0.0076 | 1.2302 |
| 828 | IK | 2018 | April | 0.0531 | 1.4817 |
| 829 | IK | 2018 | April | 0.0840 | 1.5014 |
| 830 | IK | 2018 | April | 0.0285 | 1.3076 |
| 831 | IK | 2018 | April | 0.0043 | 1.1892 |
| 832 | IK | 2018 | April | 0.0228 | 1.4288 |
| 833 | IK | 2018 | April | 0.0217 | 1.4660 |
| 834 | IK | 2019 | April | 0.1044 | 2.0389 |
| 835 | IK | 2019 | April | 0.1101 | 2.1117 |
| 836 | IK | 2019 | April | 0.1165 | 2.1457 |
| 837 | IK | 2019 | April | 0.1139 | 2.1318 |
| 838 | IK | 2019 | April | 0.1113 | 2.1090 |
| 839 | IK | 2019 | April | 0.1182 | 2.1424 |
| 840 | IK | 2019 | April | 0.1076 | 2.0956 |
| 841 | IK | 2019 | April | 0.1279 | 2.1799 |
| 842 | IK | 2019 | April | 0.1152 | 2.0953 |
| 843 | IK | 2019 | April | 0.1300 | 2.1644 |
| 844 | IK | 2019 | April | 0.2002 | 2.2115 |
| 845 | IK | 2019 | April | 0.2243 | 2.3023 |
| 846 | IK | 2019 | April | 0.2202 | 2.2566 |
| 847 | IK | 2019 | April | 0.2422 | 2.2544 |
| 848 | IK | 2019 | April | 0.2048 | 2.2305 |
| 849 | IK | 2019 | April | 0.2659 | 2.2333 |
| 850 | IK | 2019 | April | 0.1848 | 2.3007 |
| 851 | IK | 2019 | April | 0.2598 | 2.3089 |
| 852 | IK | 2019 | April | 0.2107 | 2.3066 |
| 853 | IK | 2019 | April | 0.2896 | 2.2820 |
| 854 | IK | 2019 | April | 0.1479 | 2.2383 |
| 855 | IK | 2019 | April | 0.1657 | 2.1985 |
| 856 | IK | 2019 | April | 0.1555 | 2.1984 |
| 857 | IK | 2019 | April | 0.1552 | 2.1482 |
| 858 | IK | 2019 | April | 0.1497 | 2.1994 |
| 859 | IK | 2019 | April | 0.2512 | 2.4152 |
| 860 | IK | 2019 | April | 0.2178 | 2.2028 |
| 861 | IK | 2019 | April | 0.3399 | 2.5144 |
| 862 | IK | 2019 | April | 0.3530 | 2.4285 |
| 863 | IK | 2019 | April | 0.1975 | 2.1668 |
| 864 | IK | 2019 | April | 0.1419 | 2.2155 |
| 865 | IK | 2019 | April | 0.1295 | 2.1647 |
| 866 | IK | 2019 | April | 0.1593 | 2.2492 |
| 867 | IK | 2019 | April | 0.1360 | 2.1661 |
| 868 | IK | 2019 | April | 0.1516 | 2.1917 |
| 869 | IK | 2019 | April | 0.1399 | 2.1514 |
| 870 | IK | 2019 | April | 0.1344 | 2.1386 |
| 871 | IK | 2019 | April | 0.1672 | 2.1807 |
| 872 | IK | 2019 | April | 0.1679 | 2.1714 |
| 873 | IK | 2019 | April | 0.1340 | 2.0755 |
| 874 | IK | 2019 | April | 0.2007 | 2.2984 |
| 875 | IK | 2019 | April | 0.2418 | 2.3037 |
| 876 | IK | 2019 | April | 0.1973 | 2.2811 |
| 877 | IK | 2019 | April | 0.3185 | 2.2817 |
| 878 | IK | 2019 | April | 0.3995 | 2.2930 |
| 879 | IK | 2019 | April | 0.3025 | 2.3143 |
| 880 | IK | 2019 | April | 0.3354 | 2.1381 |
| 881 | IK | 2019 | April | 0.4119 | 1.9502 |
| 882 | IK | 2019 | April | 0.5981 | 1.9524 |
| 883 | IK | 2019 | April | 0.7817 | 1.5310 |
| 884 | IK | 2019 | April | 0.4031 | 2.2689 |
| 885 | IK | 2019 | April | 0.2578 | 2.0591 |
| 886 | IK | 2019 | April | 0.2786 | 2.0984 |
| 887 | IK | 2019 | April | 0.5170 | 2.1457 |
| 888 | IK | 2019 | April | 0.1798 | 2.2494 |
| 889 | IK | 2019 | April | 0.4122 | 2.0953 |
| 890 | IK | 2019 | April | 0.5942 | 1.9309 |
| 891 | IK | 2019 | April | 0.3475 | 2.2923 |
| 892 | IK | 2019 | April | 0.4788 | 2.2579 |
| 893 | IK | 2019 | April | 0.4814 | 2.1886 |
| 894 | IK | 2019 | April | 0.3185 | 2.2935 |
| 895 | IK | 2019 | April | 0.3071 | 2.2834 |
| 896 | IK | 2019 | April | 0.2647 | 2.2562 |
| 897 | IK | 2019 | April | 0.3861 | 2.2242 |
| 898 | IK | 2019 | April | 0.3947 | 2.2071 |
| 899 | IK | 2019 | April | 0.1862 | 2.2918 |
| 900 | IK | 2019 | April | 0.2052 | 2.2652 |
| 901 | IK | 2019 | April | 0.2498 | 2.2529 |
| 902 | IK | 2019 | April | 0.3638 | 2.2259 |
| 903 | IK | 2019 | April | 0.4059 | 2.1518 |
| 904 | IK | 2019 | April | 0.2794 | 2.5939 |
| 905 | IK | 2019 | April | 0.4749 | 2.2686 |
| 906 | IK | 2019 | April | 0.4148 | 2.3762 |
| 907 | IK | 2019 | April | 0.3391 | 2.4658 |
| 908 | IK | 2019 | April | 0.3058 | 2.4954 |
| 909 | IK | 2019 | April | 0.2720 | 2.3651 |
| 910 | IK | 2019 | April | 0.1965 | 2.2810 |
| 911 | IK | 2019 | April | 0.2659 | 2.3306 |
| 912 | IK | 2019 | April | 0.4924 | 2.1394 |
| 913 | IK | 2019 | April | 0.3212 | 2.1209 |
| 914 | IK | 2019 | April | 0.4316 | 2.2075 |
| 915 | IK | 2019 | April | 0.3832 | 2.3332 |
| 916 | IK | 2019 | April | 0.2631 | 2.2365 |
| 917 | IK | 2019 | April | 0.3013 | 2.3297 |
| 918 | IK | 2019 | April | 0.6238 | 1.7900 |
| 919 | IK | 2019 | April | 0.6338 | 1.7948 |
| 920 | IK | 2019 | April | 0.5642 | 2.1255 |
| 921 | IK | 2019 | April | 0.3993 | 2.3352 |
| 922 | IK | 2019 | April | 0.5923 | 1.9785 |
| 923 | IK | 2019 | April | 0.5892 | 1.9805 |
| 924 | IK | 2019 | April | 0.2600 | 2.4187 |
| 925 | IK | 2019 | April | 0.2397 | 2.3951 |
| 926 | IK | 2019 | April | 0.3122 | 2.3362 |
| 927 | IK | 2019 | April | 0.5899 | 2.0292 |
| 928 | IK | 2019 | April | 0.3658 | 2.4591 |
| 929 | IK | 2019 | April | 0.5084 | 2.2497 |
| 930 | IK | 2019 | April | 0.6620 | 1.5625 |
| 931 | IK | 2019 | April | 0.6121 | 1.8852 |
| 932 | IK | 2019 | April | 0.6615 | 1.5977 |
| 933 | IK | 2019 | April | 0.6485 | 1.7427 |
| 934 | IK | 2019 | April | 0.2023 | 2.2964 |
| 935 | IK | 2019 | April | 0.1962 | 2.1036 |
| 936 | IK | 2019 | April | 0.2910 | 2.3859 |
| 937 | IK | 2019 | April | 0.2673 | 2.4293 |
| 938 | IK | 2019 | April | 0.2333 | 2.4121 |
| 939 | IK | 2019 | April | 0.1790 | 2.1372 |
| 940 | IK | 2019 | April | 0.1716 | 2.1745 |
| 941 | IK | 2019 | April | 0.1658 | 2.2095 |
| 942 | IK | 2019 | April | 0.1304 | 2.0622 |
| 943 | IK | 2019 | April | 0.1179 | 2.0760 |
| 944 | IK | 2019 | April | 0.2577 | 2.3335 |
| 945 | IK | 2019 | April | 0.4338 | 2.1313 |
| 946 | IK | 2019 | April | 0.5148 | 2.0135 |
| 947 | IK | 2019 | April | 0.3308 | 2.2124 |
| 948 | IK | 2019 | April | 0.2776 | 2.1931 |
| 949 | IK | 2019 | April | 0.3940 | 2.2152 |
| 950 | IK | 2019 | April | 0.3801 | 2.1854 |
| 951 | IK | 2019 | April | 0.2780 | 2.2673 |
| 952 | IK | 2019 | April | 0.1828 | 2.2189 |
| 953 | IK | 2019 | April | 0.2004 | 2.2815 |
| 954 | IK | 2019 | April | 0.1707 | 2.2190 |
| 955 | IK | 2019 | April | 0.1651 | 2.2235 |
| 956 | IK | 2019 | April | 0.1668 | 2.2007 |
| 957 | IK | 2019 | April | 0.1656 | 2.1960 |
| 958 | IK | 2019 | April | 0.1858 | 2.2098 |
| 959 | IK | 2019 | April | 0.1701 | 2.1528 |
| 960 | IK | 2019 | April | 0.5038 | 1.9823 |
| 961 | IK | 2019 | April | 0.4895 | 2.0029 |
| 962 | IK | 2019 | April | 0.1775 | 2.1996 |
| 963 | IK | 2019 | April | 0.1968 | 2.2303 |
| 964 | IK | 2018 | December | 0.5585 | 1.6882 |
| 965 | IK | 2018 | December | 0.2706 | 1.9698 |
| 966 | IK | 2018 | December | 0.4526 | 1.8519 |
| 967 | IK | 2018 | December | 0.6322 | 1.6357 |
| 968 | IK | 2018 | December | 0.7716 | 1.3071 |
| 969 | IK | 2018 | December | 0.9376 | 0.7129 |
| 970 | IK | 2018 | December | 0.9174 | 0.6466 |
| 971 | IK | 2018 | December | 0.8964 | 0.9746 |
| 972 | IK | 2018 | December | 0.8446 | 1.1114 |
| 973 | IK | 2018 | December | 0.8983 | 0.7493 |
| 974 | IK | 2018 | December | 0.6038 | 1.9390 |
| 975 | IK | 2018 | December | 0.2325 | 1.9926 |
| 976 | IK | 2018 | December | 0.3339 | 1.8492 |
| 977 | IK | 2018 | December | 0.9811 | 0.4500 |
| 978 | IK | 2018 | December | 0.9833 | 0.4411 |
| 979 | IK | 2018 | December | 0.9866 | 0.3788 |
| 980 | IK | 2018 | December | 0.9906 | 0.3403 |
| 981 | IK | 2018 | December | 0.9394 | 0.8365 |
| 982 | IK | 2018 | December | 0.6240 | 1.8676 |
| 983 | IK | 2018 | December | 0.6394 | 1.8928 |
| 984 | IK | 2018 | December | 0.3204 | 1.5811 |
| 985 | IK | 2018 | December | 0.5898 | 1.8806 |
| 986 | IK | 2018 | December | 0.7758 | 1.3444 |
| 987 | IK | 2018 | December | 0.8704 | 1.0080 |
| 988 | IK | 2018 | December | 0.7753 | 1.3437 |
| 989 | IK | 2018 | December | 0.3754 | 2.0173 |
| 990 | IK | 2018 | December | 0.9516 | 0.6489 |
| 991 | IK | 2018 | December | 0.9343 | 0.7772 |
| 992 | IK | 2018 | December | 0.8893 | 1.0652 |
| 993 | IK | 2018 | December | 0.7728 | 1.3259 |
| 994 | IK | 2018 | December | 0.7363 | 1.0949 |
| 995 | IK | 2018 | December | 0.4353 | 1.0115 |
| 996 | IK | 2018 | December | 0.5564 | 1.0867 |
| 997 | IK | 2018 | December | 0.5878 | 1.8410 |
| 998 | IK | 2018 | December | 0.4875 | 2.0319 |
| 999 | IK | 2018 | December | 0.6252 | 1.8770 |
| 1000 | IK | 2018 | December | 0.6353 | 1.7286 |
| 1001 | IK | 2018 | December | 0.6469 | 1.5652 |
| 1002 | IK | 2018 | December | 0.8069 | 1.4545 |
| 1003 | IK | 2018 | December | 0.7542 | 1.5119 |
| 1004 | IK | 2018 | December | 0.1777 | 1.9147 |
| 1005 | IK | 2018 | December | 0.6524 | 1.9127 |
| 1006 | IK | 2018 | December | 0.2108 | 1.5612 |
| 1007 | IK | 2018 | December | 0.2170 | 1.6285 |
| 1008 | IK | 2018 | December | 0.4855 | 1.9324 |
| 1009 | IK | 2018 | December | 0.6689 | 1.5754 |
| 1010 | IK | 2018 | December | 0.2908 | 1.8461 |
| 1011 | IK | 2018 | December | 0.6228 | 1.7571 |
| 1012 | IK | 2018 | December | 0.6533 | 1.5257 |
| 1013 | IK | 2018 | December | 0.3255 | 2.2181 |
| 1014 | IK | 2018 | December | 0.9036 | 0.8903 |
| 1015 | IK | 2018 | December | 0.2268 | 1.3439 |
| 1016 | IK | 2018 | December | 0.3402 | 1.3734 |
| 1017 | IK | 2018 | December | 0.9789 | 0.4076 |
| 1018 | IK | 2018 | December | 0.9372 | 0.5861 |
| 1019 | IK | 2018 | December | 0.7496 | 1.0637 |
| 1020 | IK | 2018 | December | 0.6158 | 1.5786 |
| 1021 | IK | 2018 | December | 0.7056 | 1.2564 |
| 1022 | IK | 2018 | December | 0.3095 | 1.2210 |
| 1023 | IK | 2018 | December | 0.7393 | 1.0749 |
| 1024 | IK | 2018 | December | 0.3526 | 1.8505 |
| 1025 | IK | 2018 | December | 0.3025 | 1.8903 |
| 1026 | IK | 2018 | December | 0.3707 | 1.9045 |
| 1027 | IK | 2018 | December | 0.9362 | 0.7309 |
| 1028 | IK | 2018 | December | 0.9982 | 0.1417 |
| 1029 | IK | 2018 | December | 0.6666 | 1.6959 |
| 1030 | IK | 2018 | December | 0.9820 | 0.4315 |
| 1031 | IK | 2018 | December | 0.9952 | 0.2060 |
| 1032 | IK | 2018 | December | 0.9322 | 0.7339 |
| 1033 | IK | 2018 | December | 0.8030 | 0.9973 |
| 1034 | IK | 2018 | December | 0.4493 | 2.0443 |
| 1035 | IK | 2018 | December | 0.1280 | 2.1442 |
| 1036 | IK | 2018 | December | 0.1119 | 2.1904 |
| 1037 | IK | 2018 | December | 0.1915 | 1.3234 |
| 1038 | IK | 2018 | December | 0.5358 | 1.8308 |
| 1039 | IK | 2018 | December | 0.4653 | 1.9028 |
| 1040 | IK | 2018 | December | 0.2199 | 2.2398 |
| 1041 | IK | 2018 | December | 0.1594 | 2.0878 |
| 1042 | IK | 2018 | December | 0.6414 | 0.9689 |
| 1043 | IK | 2018 | December | 0.4950 | 1.0475 |
| 1044 | IK | 2018 | December | 0.7855 | 1.7185 |
| 1045 | IK | 2018 | December | 0.7790 | 1.7618 |
| 1046 | IK | 2018 | December | 0.4967 | 2.1338 |
| 1047 | IK | 2018 | December | 0.7658 | 1.8306 |
| 1048 | IK | 2018 | December | 0.8609 | 1.4607 |
| 1049 | IK | 2018 | December | 0.9954 | 0.2430 |
| 1050 | IK | 2018 | December | 0.9311 | 0.9340 |
| 1051 | IK | 2018 | December | 0.7766 | 1.0023 |
| 1052 | IK | 2018 | December | 0.4993 | 1.1499 |
| 1053 | IK | 2018 | December | 0.6001 | 1.2650 |
| 1054 | IK | 2018 | December | 0.3822 | 2.1341 |
| 1055 | IK | 2018 | December | 0.1355 | 1.4857 |
| 1056 | IK | 2018 | December | 0.3931 | 2.0284 |
| 1057 | IK | 2018 | December | 0.2130 | 1.3567 |
| 1058 | IK | 2018 | December | 0.0832 | 1.1798 |
| 1059 | IK | 2018 | December | 0.0613 | 1.2598 |
| 1060 | IK | 2018 | December | 0.0189 | 1.0308 |
| 1061 | IK | 2018 | December | 0.4035 | 1.5381 |
| 1062 | IK | 2018 | December | 0.0051 | 1.0113 |
| 1063 | IK | 2018 | December | 0.0432 | 1.0857 |
| 1064 | IK | 2018 | December | 0.5866 | 1.8712 |
| 1065 | IK | 2018 | December | 0.7573 | 1.4354 |
| 1066 | IK | 2018 | December | 0.6763 | 1.5839 |
| 1067 | IK | 2018 | December | 0.6150 | 1.6095 |
| 1068 | IK | 2018 | December | 0.9073 | 0.8790 |
| 1069 | IK | 2018 | December | 0.9374 | 0.7607 |
| 1070 | IK | 2018 | December | 0.7688 | 1.1845 |
| 1071 | IK | 2018 | December | 0.6728 | 1.3505 |
| 1072 | IK | 2018 | December | 0.6716 | 1.8544 |
| 1073 | IK | 2018 | December | 0.5736 | 1.9019 |
| 1074 | IK | 2018 | December | 0.8686 | 1.1250 |
| 1075 | IK | 2018 | December | 0.4488 | 1.8488 |
| 1076 | IK | 2018 | December | 0.3065 | 1.4348 |
| 1077 | IK | 2018 | December | 0.6629 | 1.6002 |
| 1078 | IK | 2018 | December | 0.5177 | 1.7603 |
| 1079 | IK | 2018 | December | 0.2050 | 1.7755 |
| 1080 | IK | 2018 | December | 0.1354 | 1.9138 |
| 1081 | IK | 2018 | December | 0.0676 | 1.6499 |
| 1082 | IK | 2018 | December | 0.2135 | 1.6215 |
| 1083 | IK | 2018 | December | 0.2491 | 1.7238 |
| 1084 | IK | 2018 | December | 0.0051 | 1.0365 |
| 1085 | IK | 2018 | December | 0.0157 | 1.0522 |
| 1086 | IK | 2018 | December | 0.0081 | 1.0592 |
| 1087 | IK | 2018 | December | 0.0319 | 1.0364 |
| 1088 | IK | 2018 | December | 0.0289 | 1.0425 |
| 1089 | IK | 2018 | December | 0.0632 | 1.0531 |
| 1090 | IK | 2018 | December | 0.1748 | 1.1483 |
| 1091 | IK | 2018 | December | 0.0400 | 1.3534 |
| 1092 | IK | 2018 | December | 0.0121 | 1.0408 |
| 1093 | IK | 2018 | December | 0.0103 | 1.0436 |
| 1094 | IK | 2019 | December | 0.4473 | 1.6249 |
| 1095 | IK | 2019 | December | 0.5233 | 1.5184 |
| 1096 | IK | 2019 | December | 0.4180 | 1.6478 |
| 1097 | IK | 2019 | December | 0.3624 | 1.5790 |
| 1098 | IK | 2019 | December | 0.3249 | 1.7209 |
| 1099 | IK | 2019 | December | 0.3385 | 1.7105 |
| 1100 | IK | 2019 | December | 0.3301 | 1.6496 |
| 1101 | IK | 2019 | December | 0.2928 | 1.8013 |
| 1102 | IK | 2019 | December | 0.3993 | 1.7176 |
| 1103 | IK | 2019 | December | 0.6254 | 1.4147 |
| 1104 | IK | 2019 | December | 0.9722 | 0.6176 |
| 1105 | IK | 2019 | December | 0.9647 | 0.6752 |
| 1106 | IK | 2019 | December | 0.9505 | 0.7178 |
| 1107 | IK | 2019 | December | 0.8626 | 1.3079 |
| 1108 | IK | 2019 | December | 0.8217 | 1.3866 |
| 1109 | IK | 2019 | December | 0.8086 | 1.4875 |
| 1110 | IK | 2019 | December | 0.9722 | 0.4867 |
| 1111 | IK | 2019 | December | 0.9586 | 0.5634 |
| 1112 | IK | 2019 | December | 0.8781 | 1.2438 |
| 1113 | IK | 2019 | December | 0.6860 | 1.6911 |
| 1114 | IK | 2019 | December | 0.9907 | 0.3600 |
| 1115 | IK | 2019 | December | 0.8334 | 1.6302 |
| 1116 | IK | 2019 | December | 0.9645 | 0.7636 |
| 1117 | IK | 2019 | December | 0.8335 | 1.6120 |
| 1118 | IK | 2019 | December | 0.7716 | 1.8103 |
| 1119 | IK | 2019 | December | 0.8247 | 1.6276 |
| 1120 | IK | 2019 | December | 0.8817 | 1.3755 |
| 1121 | IK | 2019 | December | 0.8561 | 1.4570 |
| 1122 | IK | 2019 | December | 0.8468 | 1.3454 |
| 1123 | IK | 2019 | December | 0.5378 | 1.7570 |
| 1124 | IK | 2019 | December | 0.5398 | 2.0776 |
| 1125 | IK | 2019 | December | 0.5208 | 1.8982 |
| 1126 | IK | 2019 | December | 0.7709 | 1.6229 |
| 1127 | IK | 2019 | December | 0.7114 | 1.8860 |
| 1128 | IK | 2019 | December | 0.7464 | 1.6937 |
| 1129 | IK | 2019 | December | 0.7014 | 1.9385 |
| 1130 | IK | 2019 | December | 0.5664 | 2.0614 |
| 1131 | IK | 2019 | December | 0.6197 | 2.1767 |
| 1132 | IK | 2019 | December | 0.6702 | 1.9818 |
| 1133 | IK | 2019 | December | 0.8661 | 1.4441 |
| 1134 | IK | 2019 | December | 0.7709 | 1.8550 |
| 1135 | IK | 2019 | December | 0.9136 | 1.2009 |
| 1136 | IK | 2019 | December | 0.8574 | 1.5285 |
| 1137 | IK | 2019 | December | 0.5566 | 2.2506 |
| 1138 | IK | 2019 | December | 0.2078 | 1.5887 |
| 1139 | IK | 2019 | December | 0.1881 | 1.5981 |
| 1140 | IK | 2019 | December | 0.0604 | 1.1926 |
| 1141 | IK | 2019 | December | 0.2822 | 2.2590 |
| 1142 | IK | 2019 | December | 0.2560 | 1.7144 |
| 1143 | IK | 2019 | December | 0.2751 | 1.7763 |
| 1144 | IK | 2019 | December | 0.6511 | 2.1546 |
| 1145 | IK | 2019 | December | 0.7356 | 2.0045 |
| 1146 | IK | 2019 | December | 0.1741 | 1.6321 |
| 1147 | IK | 2019 | December | 0.6854 | 2.1695 |
| 1148 | IK | 2019 | December | 0.7445 | 1.9483 |
| 1149 | IK | 2019 | December | 0.3855 | 2.0082 |
| 1150 | IK | 2019 | December | 0.2846 | 1.9441 |
| 1151 | IK | 2019 | December | 0.1860 | 2.1517 |
| 1152 | IK | 2019 | December | 0.2970 | 2.3340 |
| 1153 | IK | 2019 | December | 0.3364 | 2.4980 |
| 1154 | IK | 2019 | December | 0.5989 | 2.0325 |
| 1155 | IK | 2019 | December | 0.8175 | 1.4744 |
| 1156 | IK | 2019 | December | 0.8373 | 1.2227 |
| 1157 | IK | 2019 | December | 0.9227 | 1.0701 |
| 1158 | IK | 2019 | December | 0.7982 | 1.4582 |
| 1159 | IK | 2019 | December | 0.8654 | 1.3301 |
| 1160 | IK | 2019 | December | 0.2035 | 2.0386 |
| 1161 | IK | 2019 | December | 0.9278 | 0.7499 |
| 1162 | IK | 2019 | December | 0.6711 | 1.6131 |
| 1163 | IK | 2019 | December | 0.9627 | 0.5867 |
| 1164 | IK | 2019 | December | 0.6927 | 2.1288 |
| 1165 | IK | 2019 | December | 0.7390 | 1.9747 |
| 1166 | IK | 2019 | December | 0.6030 | 2.1755 |
| 1167 | IK | 2019 | December | 0.8284 | 1.5973 |
| 1168 | IK | 2019 | December | 0.8129 | 1.4492 |
| 1169 | IK | 2019 | December | 0.7824 | 1.7777 |
| 1170 | IK | 2019 | December | 0.7924 | 1.6841 |
| 1171 | IK | 2019 | December | 0.7759 | 1.7809 |
| 1172 | IK | 2019 | December | 0.9385 | 1.0169 |
| 1173 | IK | 2019 | December | 0.8281 | 1.6535 |
| 1174 | IK | 2019 | December | 0.9162 | 0.9202 |
| 1175 | IK | 2019 | December | 0.9144 | 1.2003 |
| 1176 | IK | 2019 | December | 0.9348 | 1.0351 |
| 1177 | IK | 2019 | December | 0.9623 | 0.7827 |
| 1178 | IK | 2019 | December | 0.9697 | 0.7149 |
| 1179 | IK | 2019 | December | 0.9486 | 0.7915 |
| 1180 | IK | 2019 | December | 0.8975 | 1.1303 |
| 1181 | IK | 2019 | December | 0.9821 | 0.5340 |
| 1182 | IK | 2019 | December | 0.7410 | 1.1590 |
| 1183 | IK | 2019 | December | 0.7530 | 1.4005 |
| 1184 | IK | 2019 | December | 0.7070 | 2.0877 |
| 1185 | IK | 2019 | December | 0.4062 | 1.9877 |
| 1186 | IK | 2019 | December | 0.6137 | 2.2856 |
| 1187 | IK | 2019 | December | 0.5792 | 2.2376 |
| 1188 | IK | 2019 | December | 0.6198 | 2.2699 |
| 1189 | IK | 2019 | December | 0.7132 | 2.0654 |
| 1190 | IK | 2019 | December | 0.7264 | 2.0095 |
| 1191 | IK | 2019 | December | 0.6842 | 1.6141 |
| 1192 | IK | 2019 | December | 0.9096 | 1.2058 |
| 1193 | IK | 2019 | December | 0.8699 | 1.2139 |
| 1194 | IK | 2019 | December | 0.9387 | 0.8260 |
| 1195 | IK | 2019 | December | 0.7328 | 1.7402 |
| 1196 | IK | 2019 | December | 0.8326 | 1.5436 |
| 1197 | IK | 2019 | December | 0.8787 | 1.2992 |
| 1198 | IK | 2019 | December | 0.7494 | 1.7317 |
| 1199 | IK | 2019 | December | 0.1804 | 2.3528 |
| 1200 | IK | 2019 | December | 0.4781 | 1.9293 |
| 1201 | IK | 2019 | December | 0.5152 | 1.9843 |
| 1202 | IK | 2019 | December | 0.7563 | 1.9176 |
| 1203 | IK | 2019 | December | 0.8953 | 1.2354 |
| 1204 | IK | 2019 | December | 0.6896 | 1.9273 |
| 1205 | IK | 2019 | December | 0.4899 | 1.9511 |
| 1206 | IK | 2019 | December | 0.4860 | 2.0763 |
| 1207 | IK | 2019 | December | 0.4798 | 1.8850 |
| 1208 | IK | 2019 | December | 0.5758 | 1.7958 |
| 1209 | IK | 2019 | December | 0.9457 | 0.7842 |
| 1210 | IK | 2019 | December | 0.7171 | 1.7017 |
| 1211 | IK | 2019 | December | 0.9652 | 0.6564 |
| 1212 | IK | 2019 | December | 0.6814 | 1.7895 |
| 1213 | IK | 2019 | December | 0.8982 | 1.0400 |
| 1214 | IK | 2019 | December | 0.1991 | 2.2739 |
| 1215 | IK | 2019 | December | 0.1061 | 2.2713 |
| 1216 | IK | 2019 | December | 0.2359 | 2.3066 |
| 1217 | IK | 2019 | December | 0.1395 | 2.0828 |
| 1218 | IK | 2019 | December | 0.2218 | 2.0021 |
| 1219 | IK | 2019 | December | 0.7884 | 1.0028 |
| 1220 | IK | 2019 | December | 0.9380 | 0.8672 |
| 1221 | IK | 2019 | December | 0.7993 | 1.4044 |
| 1222 | IK | 2019 | December | 0.2199 | 2.1444 |
| 1223 | IK | 2019 | December | 0.2553 | 2.1921 |
| 1224 | HPM | 2018 | April | 0.3845 | 1.4501 |
| 1225 | HPM | 2018 | April | 0.0948 | 1.0552 |
| 1226 | HPM | 2018 | April | 0.2330 | 1.1497 |
| 1227 | HPM | 2018 | April | 0.6145 | 1.4171 |
| 1228 | HPM | 2018 | April | 0.8124 | 0.9320 |
| 1229 | HPM | 2018 | April | 0.7408 | 0.9723 |
| 1230 | HPM | 2018 | April | 0.1994 | 1.1382 |
| 1231 | HPM | 2018 | April | 0.6886 | 1.0460 |
| 1232 | HPM | 2018 | April | 0.4839 | 1.3240 |
| 1233 | HPM | 2018 | April | 0.0814 | 1.0763 |
| 1234 | HPM | 2018 | April | 0.5767 | 1.7342 |
| 1235 | HPM | 2018 | April | 0.9723 | 0.3278 |
| 1236 | HPM | 2018 | April | 0.6976 | 1.2901 |
| 1237 | HPM | 2018 | April | 0.9335 | 0.8253 |
| 1238 | HPM | 2018 | April | 0.9223 | 0.8790 |
| 1239 | HPM | 2018 | April | 0.7503 | 1.4021 |
| 1240 | HPM | 2018 | April | 0.8809 | 1.0289 |
| 1241 | HPM | 2018 | April | 0.5926 | 1.7065 |
| 1242 | HPM | 2018 | April | 0.8344 | 1.2636 |
| 1243 | HPM | 2018 | April | 0.8549 | 1.1668 |
| 1244 | HPM | 2018 | April | 0.9166 | 0.8956 |
| 1245 | HPM | 2018 | April | 0.8948 | 1.0481 |
| 1246 | HPM | 2018 | April | 0.8252 | 1.2860 |
| 1247 | HPM | 2018 | April | 0.6155 | 1.1627 |
| 1248 | HPM | 2018 | April | 0.7829 | 1.3791 |
| 1249 | HPM | 2018 | April | 0.4551 | 1.5716 |
| 1250 | HPM | 2018 | April | 0.7099 | 1.4981 |
| 1251 | HPM | 2018 | April | 0.7945 | 1.3138 |
| 1252 | HPM | 2018 | April | 0.7446 | 1.4327 |
| 1253 | HPM | 2018 | April | 0.1723 | 1.3225 |
| 1254 | HPM | 2018 | April | 0.2397 | 2.0435 |
| 1255 | HPM | 2018 | April | 0.7604 | 1.5582 |
| 1256 | HPM | 2018 | April | 0.8479 | 1.2200 |
| 1257 | HPM | 2018 | April | 0.6129 | 1.8213 |
| 1258 | HPM | 2018 | April | 0.7377 | 1.4914 |
| 1259 | HPM | 2018 | April | 0.8716 | 1.0010 |
| 1260 | HPM | 2018 | April | 0.6086 | 1.8643 |
| 1261 | HPM | 2018 | April | 0.6553 | 1.6403 |
| 1262 | HPM | 2018 | April | 0.8812 | 1.0564 |
| 1263 | HPM | 2018 | April | 0.3969 | 1.8363 |
| 1264 | HPM | 2018 | April | 0.9216 | 0.8350 |
| 1265 | HPM | 2018 | April | 0.9088 | 0.5793 |
| 1266 | HPM | 2018 | April | 0.9795 | 0.4444 |
| 1267 | HPM | 2018 | April | 0.7247 | 1.2705 |
| 1268 | HPM | 2018 | April | 0.8118 | 1.3879 |
| 1269 | HPM | 2018 | April | 0.8965 | 0.9210 |
| 1270 | HPM | 2018 | April | 0.9119 | 0.8397 |
| 1271 | HPM | 2018 | April | 0.9309 | 0.7481 |
| 1272 | HPM | 2018 | April | 0.6426 | 1.0464 |
| 1273 | HPM | 2018 | April | 0.9141 | 0.8343 |
| 1274 | HPM | 2018 | April | 0.9305 | 0.8380 |
| 1275 | HPM | 2018 | April | 0.8368 | 1.1582 |
| 1276 | HPM | 2018 | April | 0.9507 | 0.5813 |
| 1277 | HPM | 2018 | April | 0.9649 | 0.6077 |
| 1278 | HPM | 2018 | April | 0.8685 | 1.1729 |
| 1279 | HPM | 2018 | April | 0.9164 | 1.0659 |
| 1280 | HPM | 2018 | April | 0.9596 | 0.5558 |
| 1281 | HPM | 2018 | April | 0.2710 | 1.4048 |
| 1282 | HPM | 2018 | April | 0.8870 | 0.9413 |
| 1283 | HPM | 2018 | April | 0.9488 | 0.6652 |
| 1284 | HPM | 2018 | April | 0.9541 | 0.6756 |
| 1285 | HPM | 2018 | April | 0.9558 | 0.5719 |
| 1286 | HPM | 2018 | April | 0.9298 | 0.7544 |
| 1287 | HPM | 2018 | April | 0.9441 | 0.7063 |
| 1288 | HPM | 2018 | April | 0.8777 | 1.1583 |
| 1289 | HPM | 2018 | April | 0.8352 | 1.2168 |
| 1290 | HPM | 2018 | April | 0.7524 | 1.4319 |
| 1291 | HPM | 2018 | April | 0.8623 | 1.1612 |
| 1292 | HPM | 2018 | April | 0.8952 | 1.0668 |
| 1293 | HPM | 2018 | April | 0.6638 | 1.2099 |
| 1294 | HPM | 2018 | April | 0.8925 | 1.1339 |
| 1295 | HPM | 2018 | April | 0.8207 | 1.4323 |
| 1296 | HPM | 2018 | April | 0.9093 | 0.9103 |
| 1297 | HPM | 2018 | April | 0.9223 | 0.9259 |
| 1298 | HPM | 2018 | April | 0.8717 | 1.1776 |
| 1299 | HPM | 2018 | April | 0.8203 | 1.4549 |
| 1300 | HPM | 2018 | April | 0.9642 | 0.5339 |
| 1301 | HPM | 2018 | April | 0.8710 | 1.2203 |
| 1302 | HPM | 2018 | April | 0.9226 | 0.8354 |
| 1303 | HPM | 2018 | April | 0.8881 | 1.0071 |
| 1304 | HPM | 2018 | April | 0.9452 | 0.7897 |
| 1305 | HPM | 2018 | April | 0.9521 | 0.6061 |
| 1306 | HPM | 2018 | April | 0.9311 | 0.8873 |
| 1307 | HPM | 2018 | April | 0.9210 | 0.9175 |
| 1308 | HPM | 2018 | April | 0.9186 | 0.8490 |
| 1309 | HPM | 2018 | April | 0.7865 | 1.5835 |
| 1310 | HPM | 2018 | April | 0.8380 | 1.1792 |
| 1311 | HPM | 2018 | April | 0.9081 | 0.8412 |
| 1312 | HPM | 2018 | April | 0.9535 | 0.7014 |
| 1313 | HPM | 2018 | April | 0.9632 | 0.5747 |
| 1314 | HPM | 2018 | April | 0.9351 | 0.9125 |
| 1315 | HPM | 2018 | April | 0.9474 | 0.7245 |
| 1316 | HPM | 2018 | April | 0.9211 | 0.8937 |
| 1317 | HPM | 2018 | April | 0.9327 | 0.7680 |
| 1318 | HPM | 2018 | April | 0.9907 | 0.3334 |
| 1319 | HPM | 2018 | April | 0.9428 | 0.6157 |
| 1320 | HPM | 2018 | April | 0.9694 | 0.5672 |
| 1321 | HPM | 2018 | April | 0.9767 | 0.4996 |
| 1322 | HPM | 2018 | April | 0.9977 | 0.1344 |
| 1323 | HPM | 2018 | April | 0.9998 | 0.0394 |
| 1324 | HPM | 2018 | April | 0.8909 | 1.0388 |
| 1325 | HPM | 2018 | April | 0.8793 | 1.0810 |
| 1326 | HPM | 2018 | April | 0.8931 | 1.0375 |
| 1327 | HPM | 2018 | April | 0.9249 | 0.9053 |
| 1328 | HPM | 2018 | April | 0.8734 | 1.1409 |
| 1329 | HPM | 2018 | April | 0.8837 | 1.1253 |
| 1330 | HPM | 2018 | April | 0.9004 | 1.0253 |
| 1331 | HPM | 2018 | April | 0.9667 | 0.5614 |
| 1332 | HPM | 2018 | April | 0.8920 | 1.1212 |
| 1333 | HPM | 2018 | April | 0.9071 | 0.9146 |
| 1334 | HPM | 2018 | April | 0.9142 | 0.7954 |
| 1335 | HPM | 2018 | April | 0.9296 | 0.8741 |
| 1336 | HPM | 2018 | April | 0.9210 | 0.7840 |
| 1337 | HPM | 2018 | April | 0.9177 | 0.7085 |
| 1338 | HPM | 2018 | April | 0.9078 | 0.9108 |
| 1339 | HPM | 2018 | April | 0.9171 | 0.8328 |
| 1340 | HPM | 2018 | April | 0.7769 | 1.1327 |
| 1341 | HPM | 2018 | April | 0.9185 | 0.8575 |
| 1342 | HPM | 2018 | April | 0.9991 | 0.0922 |
| 1343 | HPM | 2018 | April | 0.9839 | 0.3688 |
| 1344 | HPM | 2018 | April | 0.9689 | 0.5511 |
| 1345 | HPM | 2018 | April | 0.9597 | 0.7474 |
| 1346 | HPM | 2018 | April | 0.9023 | 1.0835 |
| 1347 | HPM | 2018 | April | 0.8605 | 1.3464 |
| 1348 | HPM | 2018 | April | 0.8232 | 1.4480 |
| 1349 | HPM | 2018 | April | 0.9245 | 1.0151 |
| 1350 | HPM | 2018 | April | 0.8826 | 1.1784 |
| 1351 | HPM | 2018 | April | 0.9984 | 0.1344 |
| 1352 | HPM | 2018 | April | 0.8848 | 1.2097 |
| 1353 | HPM | 2018 | April | 0.8561 | 1.3307 |
| 1354 | HPM | 2019 | April | 0.3925 | 2.1973 |
| 1355 | HPM | 2019 | April | 0.5992 | 1.8314 |
| 1356 | HPM | 2019 | April | 0.4247 | 2.1657 |
| 1357 | HPM | 2019 | April | 0.4528 | 2.1959 |
| 1358 | HPM | 2019 | April | 0.5475 | 1.9538 |
| 1359 | HPM | 2019 | April | 0.4830 | 2.1117 |
| 1360 | HPM | 2019 | April | 0.4188 | 2.1158 |
| 1361 | HPM | 2019 | April | 0.3753 | 2.0813 |
| 1362 | HPM | 2019 | April | 0.5142 | 2.0351 |
| 1363 | HPM | 2019 | April | 0.6239 | 1.7586 |
| 1364 | HPM | 2019 | April | 0.3908 | 2.2182 |
| 1365 | HPM | 2019 | April | 0.3044 | 2.2603 |
| 1366 | HPM | 2019 | April | 0.3789 | 2.2117 |
| 1367 | HPM | 2019 | April | 0.4153 | 2.2316 |
| 1368 | HPM | 2019 | April | 0.4751 | 2.1622 |
| 1369 | HPM | 2019 | April | 0.5304 | 2.0382 |
| 1370 | HPM | 2019 | April | 0.4537 | 1.9233 |
| 1371 | HPM | 2019 | April | 0.6390 | 1.7022 |
| 1372 | HPM | 2019 | April | 0.5228 | 1.9058 |
| 1373 | HPM | 2019 | April | 0.5227 | 1.9943 |
| 1374 | HPM | 2019 | April | 0.6388 | 1.7341 |
| 1375 | HPM | 2019 | April | 0.6528 | 1.6187 |
| 1376 | HPM | 2019 | April | 0.6061 | 1.9175 |
| 1377 | HPM | 2019 | April | 0.6171 | 1.8680 |
| 1378 | HPM | 2019 | April | 0.5476 | 2.0138 |
| 1379 | HPM | 2019 | April | 0.6377 | 1.7871 |
| 1380 | HPM | 2019 | April | 0.5970 | 1.9557 |
| 1381 | HPM | 2019 | April | 0.6075 | 1.9475 |
| 1382 | HPM | 2019 | April | 0.6085 | 1.9123 |
| 1383 | HPM | 2019 | April | 0.6251 | 1.8224 |
| 1384 | HPM | 2019 | April | 0.6629 | 1.5134 |
| 1385 | HPM | 2019 | April | 0.6674 | 1.4545 |
| 1386 | HPM | 2019 | April | 0.6695 | 1.4609 |
| 1387 | HPM | 2019 | April | 0.6608 | 1.5813 |
| 1388 | HPM | 2019 | April | 0.6475 | 1.7227 |
| 1389 | HPM | 2019 | April | 0.5692 | 1.9925 |
| 1390 | HPM | 2019 | April | 0.6156 | 1.9174 |
| 1391 | HPM | 2019 | April | 0.5575 | 2.1379 |
| 1392 | HPM | 2019 | April | 0.6488 | 1.7315 |
| 1393 | HPM | 2019 | April | 0.6692 | 1.4888 |
| 1394 | HPM | 2019 | April | 0.6422 | 1.7108 |
| 1395 | HPM | 2019 | April | 0.6227 | 1.8204 |
| 1396 | HPM | 2019 | April | 0.6628 | 1.6330 |
| 1397 | HPM | 2019 | April | 0.6710 | 1.5330 |
| 1398 | HPM | 2019 | April | 0.6672 | 1.5667 |
| 1399 | HPM | 2019 | April | 0.6659 | 1.5619 |
| 1400 | HPM | 2019 | April | 0.6729 | 1.4540 |
| 1401 | HPM | 2019 | April | 0.6663 | 1.5179 |
| 1402 | HPM | 2019 | April | 0.5948 | 1.8748 |
| 1403 | HPM | 2019 | April | 0.4128 | 2.0791 |
| 1404 | HPM | 2019 | April | 0.5890 | 1.9788 |
| 1405 | HPM | 2019 | April | 0.6720 | 1.4231 |
| 1406 | HPM | 2019 | April | 0.6365 | 1.7950 |
| 1407 | HPM | 2019 | April | 0.5429 | 1.9858 |
| 1408 | HPM | 2019 | April | 0.6148 | 1.8826 |
| 1409 | HPM | 2019 | April | 0.6239 | 1.8301 |
| 1410 | HPM | 2019 | April | 0.4966 | 1.9807 |
| 1411 | HPM | 2019 | April | 0.6239 | 1.7868 |
| 1412 | HPM | 2019 | April | 0.6262 | 1.7998 |
| 1413 | HPM | 2019 | April | 0.6257 | 1.7989 |
| 1414 | HPM | 2019 | April | 0.6159 | 1.9316 |
| 1415 | HPM | 2019 | April | 0.6417 | 1.7696 |
| 1416 | HPM | 2019 | April | 0.5392 | 1.9685 |
| 1417 | HPM | 2019 | April | 0.6186 | 1.8538 |
| 1418 | HPM | 2019 | April | 0.3623 | 2.3320 |
| 1419 | HPM | 2019 | April | 0.6143 | 1.8581 |
| 1420 | HPM | 2019 | April | 0.6342 | 1.8140 |
| 1421 | HPM | 2019 | April | 0.4505 | 2.2294 |
| 1422 | HPM | 2019 | April | 0.3399 | 2.3975 |
| 1423 | HPM | 2019 | April | 0.3781 | 2.2875 |
| 1424 | HPM | 2019 | April | 0.6582 | 1.6550 |
| 1425 | HPM | 2019 | April | 0.5534 | 2.0201 |
| 1426 | HPM | 2019 | April | 0.5197 | 2.0536 |
| 1427 | HPM | 2019 | April | 0.4063 | 2.1825 |
| 1428 | HPM | 2019 | April | 0.3837 | 2.3483 |
| 1429 | HPM | 2019 | April | 0.6087 | 1.9756 |
| 1430 | HPM | 2019 | April | 0.6415 | 1.7464 |
| 1431 | HPM | 2019 | April | 0.6196 | 1.8534 |
| 1432 | HPM | 2019 | April | 0.3966 | 2.1840 |
| 1433 | HPM | 2019 | April | 0.4870 | 2.1073 |
| 1434 | HPM | 2019 | April | 0.5609 | 1.9883 |
| 1435 | HPM | 2019 | April | 0.5998 | 1.9573 |
| 1436 | HPM | 2019 | April | 0.6380 | 1.7784 |
| 1437 | HPM | 2019 | April | 0.6269 | 1.8387 |
| 1438 | HPM | 2019 | April | 0.6420 | 1.7391 |
| 1439 | HPM | 2019 | April | 0.6156 | 1.8863 |
| 1440 | HPM | 2019 | April | 0.5707 | 2.0521 |
| 1441 | HPM | 2019 | April | 0.6277 | 1.8380 |
| 1442 | HPM | 2019 | April | 0.5942 | 1.9287 |
| 1443 | HPM | 2019 | April | 0.6314 | 1.7668 |
| 1444 | HPM | 2019 | April | 0.6302 | 1.8440 |
| 1445 | HPM | 2019 | April | 0.6197 | 1.8922 |
| 1446 | HPM | 2019 | April | 0.6207 | 1.8553 |
| 1447 | HPM | 2019 | April | 0.6264 | 1.8311 |
| 1448 | HPM | 2019 | April | 0.2563 | 2.2716 |
| 1449 | HPM | 2019 | April | 0.3423 | 2.3995 |
| 1450 | HPM | 2019 | April | 0.3134 | 2.3902 |
| 1451 | HPM | 2019 | April | 0.5842 | 2.0603 |
| 1452 | HPM | 2019 | April | 0.6271 | 1.8746 |
| 1453 | HPM | 2019 | April | 0.6093 | 1.9290 |
| 1454 | HPM | 2019 | April | 0.6160 | 1.8972 |
| 1455 | HPM | 2019 | April | 0.6474 | 1.7632 |
| 1456 | HPM | 2019 | April | 0.6099 | 1.9428 |
| 1457 | HPM | 2019 | April | 0.4833 | 2.2350 |
| 1458 | HPM | 2019 | April | 0.6322 | 1.6055 |
| 1459 | HPM | 2019 | April | 0.6821 | 1.6322 |
| 1460 | HPM | 2019 | April | 0.6241 | 1.9203 |
| 1461 | HPM | 2019 | April | 0.6003 | 2.0175 |
| 1462 | HPM | 2019 | April | 0.6444 | 1.9082 |
| 1463 | HPM | 2019 | April | 0.6977 | 1.6373 |
| 1464 | HPM | 2019 | April | 0.6350 | 1.8325 |
| 1465 | HPM | 2019 | April | 0.5700 | 2.0939 |
| 1466 | HPM | 2019 | April | 0.6069 | 1.9727 |
| 1467 | HPM | 2019 | April | 0.4641 | 2.0766 |
| 1468 | HPM | 2019 | April | 0.6239 | 2.0229 |
| 1469 | HPM | 2019 | April | 0.5730 | 2.1729 |
| 1470 | HPM | 2019 | April | 0.6358 | 2.0451 |
| 1471 | HPM | 2019 | April | 0.4470 | 2.0243 |
| 1472 | HPM | 2019 | April | 0.7888 | 1.4422 |
| 1473 | HPM | 2019 | April | 0.6821 | 1.6873 |
| 1474 | HPM | 2019 | April | 0.9063 | 1.0310 |
| 1475 | HPM | 2019 | April | 0.9258 | 0.9099 |
| 1476 | HPM | 2019 | April | 0.9101 | 0.9282 |
| 1477 | HPM | 2019 | April | 0.9425 | 0.7946 |
| 1478 | HPM | 2019 | April | 0.9307 | 0.8873 |
| 1479 | HPM | 2019 | April | 0.8182 | 1.1995 |
| 1480 | HPM | 2019 | April | 0.7307 | 1.2420 |
| 1481 | HPM | 2019 | April | 0.9151 | 0.9461 |
| 1482 | HPM | 2019 | April | 0.9157 | 0.7620 |
| 1483 | HPM | 2019 | April | 0.8736 | 0.9537 |
| 1484 | HPM | 2018 | December | 0.3504 | 1.4346 |
| 1485 | HPM | 2018 | December | 0.2489 | 1.6913 |
| 1486 | HPM | 2018 | December | 0.1261 | 1.9073 |
| 1487 | HPM | 2018 | December | 0.2466 | 1.5660 |
| 1488 | HPM | 2018 | December | 0.6087 | 1.3464 |
| 1489 | HPM | 2018 | December | 0.2628 | 1.5588 |
| 1490 | HPM | 2018 | December | 0.2935 | 1.5330 |
| 1491 | HPM | 2018 | December | 0.3023 | 1.5383 |
| 1492 | HPM | 2018 | December | 0.1586 | 1.5924 |
| 1493 | HPM | 2018 | December | 0.2188 | 1.5899 |
| 1494 | HPM | 2018 | December | 0.3996 | 2.0309 |
| 1495 | HPM | 2018 | December | 0.4419 | 2.0521 |
| 1496 | HPM | 2018 | December | 0.5141 | 1.9619 |
| 1497 | HPM | 2018 | December | 0.2543 | 2.1835 |
| 1498 | HPM | 2018 | December | 0.1405 | 2.2699 |
| 1499 | HPM | 2018 | December | 0.1064 | 2.2293 |
| 1500 | HPM | 2018 | December | 0.1014 | 2.2434 |
| 1501 | HPM | 2018 | December | 0.1616 | 2.2116 |
| 1502 | HPM | 2018 | December | 0.3384 | 2.1348 |
| 1503 | HPM | 2018 | December | 0.2497 | 2.1600 |
| 1504 | HPM | 2018 | December | 0.8412 | 1.1670 |
| 1505 | HPM | 2018 | December | 0.7586 | 1.4243 |
| 1506 | HPM | 2018 | December | 0.5563 | 1.6552 |
| 1507 | HPM | 2018 | December | 0.1725 | 2.1018 |
| 1508 | HPM | 2018 | December | 0.1846 | 2.0745 |
| 1509 | HPM | 2018 | December | 0.2604 | 1.9668 |
| 1510 | HPM | 2018 | December | 0.3933 | 1.9735 |
| 1511 | HPM | 2018 | December | 0.7041 | 1.5967 |
| 1512 | HPM | 2018 | December | 0.5829 | 1.7597 |
| 1513 | HPM | 2018 | December | 0.6975 | 1.5860 |
| 1514 | HPM | 2018 | December | 0.6358 | 1.5459 |
| 1515 | HPM | 2018 | December | 0.9941 | 0.2049 |
| 1516 | HPM | 2018 | December | 0.9492 | 0.6113 |
| 1517 | HPM | 2018 | December | 0.7460 | 1.2727 |
| 1518 | HPM | 2018 | December | 0.3433 | 1.6617 |
| 1519 | HPM | 2018 | December | 0.5424 | 1.5201 |
| 1520 | HPM | 2018 | December | 0.4331 | 1.7677 |
| 1521 | HPM | 2018 | December | 0.7424 | 1.3743 |
| 1522 | HPM | 2018 | December | 0.9175 | 0.7567 |
| 1523 | HPM | 2018 | December | 0.7136 | 1.2323 |
| 1524 | HPM | 2018 | December | 0.9997 | 0.0460 |
| 1525 | HPM | 2018 | December | 0.9191 | 0.8424 |
| 1526 | HPM | 2018 | December | 0.9832 | 0.3361 |
| 1527 | HPM | 2018 | December | 0.9787 | 0.4098 |
| 1528 | HPM | 2018 | December | 0.7881 | 1.2230 |
| 1529 | HPM | 2018 | December | 0.7271 | 1.1752 |
| 1530 | HPM | 2018 | December | 0.7875 | 1.1585 |
| 1531 | HPM | 2018 | December | 0.9970 | 0.1497 |
| 1532 | HPM | 2018 | December | 0.7773 | 1.1598 |
| 1533 | HPM | 2018 | December | 0.9152 | 0.7667 |
| 1534 | HPM | 2018 | December | 0.9618 | 0.6070 |
| 1535 | HPM | 2018 | December | 0.9985 | 0.1287 |
| 1536 | HPM | 2018 | December | 0.9390 | 0.8573 |
| 1537 | HPM | 2018 | December | 0.9319 | 0.9077 |
| 1538 | HPM | 2018 | December | 0.8960 | 0.8112 |
| 1539 | HPM | 2018 | December | 0.8307 | 0.9163 |
| 1540 | HPM | 2018 | December | 0.8962 | 1.1252 |
| 1541 | HPM | 2018 | December | 0.9130 | 1.0295 |
| 1542 | HPM | 2018 | December | 0.5181 | 1.3860 |
| 1543 | HPM | 2018 | December | 0.9195 | 0.7052 |
| 1544 | HPM | 2018 | December | 0.9289 | 0.9005 |
| 1545 | HPM | 2018 | December | 0.9325 | 0.8568 |
| 1546 | HPM | 2018 | December | 0.2588 | 1.5487 |
| 1547 | HPM | 2018 | December | 0.8917 | 1.0088 |
| 1548 | HPM | 2018 | December | 0.8048 | 0.8754 |
| 1549 | HPM | 2018 | December | 0.7934 | 0.7394 |
| 1550 | HPM | 2018 | December | 0.9328 | 0.8874 |
| 1551 | HPM | 2018 | December | 0.9359 | 0.9419 |
| 1552 | HPM | 2018 | December | 0.9880 | 0.3824 |
| 1553 | HPM | 2018 | December | 0.9990 | 0.1055 |
| 1554 | HPM | 2018 | December | 0.0901 | 1.2188 |
| 1555 | HPM | 2018 | December | 0.3595 | 1.4362 |
| 1556 | HPM | 2018 | December | 0.9203 | 0.9658 |
| 1557 | HPM | 2018 | December | 0.9655 | 0.5600 |
| 1558 | HPM | 2018 | December | 0.9822 | 0.4296 |
| 1559 | HPM | 2018 | December | 0.8966 | 1.1684 |
| 1560 | HPM | 2018 | December | 0.3526 | 1.6557 |
| 1561 | HPM | 2018 | December | 0.0882 | 1.5577 |
| 1562 | HPM | 2018 | December | 0.5552 | 1.5865 |
| 1563 | HPM | 2018 | December | 0.9860 | 0.4417 |
| 1564 | HPM | 2018 | December | 0.2695 | 1.7353 |
| 1565 | HPM | 2018 | December | 0.8528 | 1.3062 |
| 1566 | HPM | 2018 | December | 0.7951 | 0.6754 |
| 1567 | HPM | 2018 | December | 0.8750 | 1.3447 |
| 1568 | HPM | 2018 | December | 0.9233 | 1.0203 |
| 1569 | HPM | 2018 | December | 0.9923 | 0.3031 |
| 1570 | HPM | 2018 | December | 0.9983 | 0.1232 |
| 1571 | HPM | 2018 | December | 0.9981 | 0.1270 |
| 1572 | HPM | 2018 | December | 0.9971 | 0.1686 |
| 1573 | HPM | 2018 | December | 0.9956 | 0.2055 |
| 1574 | HPM | 2018 | December | 0.9405 | 0.8996 |
| 1575 | HPM | 2018 | December | 0.9607 | 0.6955 |
| 1576 | HPM | 2018 | December | 0.9958 | 0.1594 |
| 1577 | HPM | 2018 | December | 0.9573 | 0.7673 |
| 1578 | HPM | 2018 | December | 0.9599 | 0.6277 |
| 1579 | HPM | 2018 | December | 0.9320 | 0.7091 |
| 1580 | HPM | 2018 | December | 0.9554 | 0.7563 |
| 1581 | HPM | 2018 | December | 0.9978 | 0.1676 |
| 1582 | HPM | 2018 | December | 0.9792 | 0.4755 |
| 1583 | HPM | 2018 | December | 0.9771 | 0.4375 |
| 1584 | HPM | 2018 | December | 0.9720 | 0.6176 |
| 1585 | HPM | 2018 | December | 0.9839 | 0.4012 |
| 1586 | HPM | 2018 | December | 0.9761 | 0.4011 |
| 1587 | HPM | 2018 | December | 0.8483 | 0.9547 |
| 1588 | HPM | 2018 | December | 0.7916 | 1.2215 |
| 1589 | HPM | 2018 | December | 0.7787 | 1.2977 |
| 1590 | HPM | 2018 | December | 0.9023 | 1.1504 |
| 1591 | HPM | 2018 | December | 0.9084 | 1.1082 |
| 1592 | HPM | 2018 | December | 0.9408 | 0.6710 |
| 1593 | HPM | 2018 | December | 0.9315 | 0.6589 |
| 1594 | HPM | 2018 | December | 0.9678 | 0.6002 |
| 1595 | HPM | 2018 | December | 0.9631 | 0.5941 |
| 1596 | HPM | 2018 | December | 0.9588 | 0.6925 |
| 1597 | HPM | 2018 | December | 0.9700 | 0.6028 |
| 1598 | HPM | 2018 | December | 0.9946 | 0.2354 |
| 1599 | HPM | 2018 | December | 0.9317 | 0.9147 |
| 1600 | HPM | 2018 | December | 0.9400 | 0.8507 |
| 1601 | HPM | 2018 | December | 0.9559 | 0.6025 |
| 1602 | HPM | 2018 | December | 0.9551 | 0.6935 |
| 1603 | HPM | 2018 | December | 0.9513 | 0.7382 |
| 1604 | HPM | 2018 | December | 0.9689 | 0.5511 |
| 1605 | HPM | 2018 | December | 0.9597 | 0.7474 |
| 1606 | HPM | 2018 | December | 0.9023 | 1.0835 |
| 1607 | HPM | 2018 | December | 0.8605 | 1.3464 |
| 1608 | HPM | 2018 | December | 0.8232 | 1.4480 |
| 1609 | HPM | 2018 | December | 0.9245 | 1.0151 |
| 1610 | HPM | 2018 | December | 0.8826 | 1.1784 |
| 1611 | HPM | 2018 | December | 0.9984 | 0.1344 |
| 1612 | HPM | 2018 | December | 0.8848 | 1.2097 |
| 1613 | HPM | 2018 | December | 0.8561 | 1.3307 |
| 1614 | HPM | 2019 | December | 0.8310 | 0.9108 |
| 1615 | HPM | 2019 | December | 0.8337 | 0.8855 |
| 1616 | HPM | 2019 | December | 0.8413 | 0.8495 |
| 1617 | HPM | 2019 | December | 0.8196 | 0.8869 |
| 1618 | HPM | 2019 | December | 0.7897 | 0.9552 |
| 1619 | HPM | 2019 | December | 0.7479 | 1.0545 |
| 1620 | HPM | 2019 | December | 0.6944 | 1.1592 |
| 1621 | HPM | 2019 | December | 0.6835 | 1.1537 |
| 1622 | HPM | 2019 | December | 0.6225 | 1.1883 |
| 1623 | HPM | 2019 | December | 0.6251 | 1.0420 |
| 1624 | HPM | 2019 | December | 0.8316 | 1.2119 |
| 1625 | HPM | 2019 | December | 0.8294 | 1.2057 |
| 1626 | HPM | 2019 | December | 0.8364 | 1.1637 |
| 1627 | HPM | 2019 | December | 0.8293 | 1.1703 |
| 1628 | HPM | 2019 | December | 0.8125 | 1.2139 |
| 1629 | HPM | 2019 | December | 0.8016 | 1.2675 |
| 1630 | HPM | 2019 | December | 0.7800 | 1.2973 |
| 1631 | HPM | 2019 | December | 0.7288 | 1.4251 |
| 1632 | HPM | 2019 | December | 0.8482 | 1.2008 |
| 1633 | HPM | 2019 | December | 0.9019 | 0.9320 |
| 1634 | HPM | 2019 | December | 0.8568 | 1.1927 |
| 1635 | HPM | 2019 | December | 0.5831 | 1.9771 |
| 1636 | HPM | 2019 | December | 0.9085 | 0.9800 |
| 1637 | HPM | 2019 | December | 0.8624 | 1.2167 |
| 1638 | HPM | 2019 | December | 0.9300 | 0.8069 |
| 1639 | HPM | 2019 | December | 0.9186 | 0.9584 |
| 1640 | HPM | 2019 | December | 0.9494 | 0.7484 |
| 1641 | HPM | 2019 | December | 0.7588 | 1.4589 |
| 1642 | HPM | 2019 | December | 0.9008 | 1.0103 |
| 1643 | HPM | 2019 | December | 0.9688 | 0.4631 |
| 1644 | HPM | 2019 | December | 0.9274 | 0.9591 |
| 1645 | HPM | 2019 | December | 0.9315 | 0.9141 |
| 1646 | HPM | 2019 | December | 0.8568 | 1.2305 |
| 1647 | HPM | 2019 | December | 0.6514 | 1.1871 |
| 1648 | HPM | 2019 | December | 0.9631 | 0.6029 |
| 1649 | HPM | 2019 | December | 0.8784 | 0.9444 |
| 1650 | HPM | 2019 | December | 0.7913 | 1.6180 |
| 1651 | HPM | 2019 | December | 0.8520 | 1.2960 |
| 1652 | HPM | 2019 | December | 0.5762 | 2.0157 |
| 1653 | HPM | 2019 | December | 0.8517 | 1.2991 |
| 1654 | HPM | 2019 | December | 0.4142 | 1.9108 |
| 1655 | HPM | 2019 | December | 0.9630 | 0.6587 |
| 1656 | HPM | 2019 | December | 0.8633 | 1.2053 |
| 1657 | HPM | 2019 | December | 0.6792 | 1.7203 |
| 1658 | HPM | 2019 | December | 0.2063 | 2.0685 |
| 1659 | HPM | 2019 | December | 0.8945 | 1.0300 |
| 1660 | HPM | 2019 | December | 0.9488 | 0.6883 |
| 1661 | HPM | 2019 | December | 0.9753 | 0.4653 |
| 1662 | HPM | 2019 | December | 0.8050 | 1.4998 |
| 1663 | HPM | 2019 | December | 0.9445 | 0.7691 |
| 1664 | HPM | 2019 | December | 0.9614 | 0.6988 |
| 1665 | HPM | 2019 | December | 0.8914 | 1.0828 |
| 1666 | HPM | 2019 | December | 0.5264 | 1.4145 |
| 1667 | HPM | 2019 | December | 0.8093 | 1.3745 |
| 1668 | HPM | 2019 | December | 0.1854 | 1.5655 |
| 1669 | HPM | 2019 | December | 0.3712 | 2.1161 |
| 1670 | HPM | 2019 | December | 0.4671 | 1.6324 |
| 1671 | HPM | 2019 | December | 0.4241 | 1.8643 |
| 1672 | HPM | 2019 | December | 0.9229 | 0.9206 |
| 1673 | HPM | 2019 | December | 0.9089 | 1.1204 |
| 1674 | HPM | 2019 | December | 0.9607 | 0.7113 |
| 1675 | HPM | 2019 | December | 0.9380 | 0.7715 |
| 1676 | HPM | 2019 | December | 0.9246 | 0.9250 |
| 1677 | HPM | 2019 | December | 0.7034 | 1.5567 |
| 1678 | HPM | 2019 | December | 0.1483 | 2.1699 |
| 1679 | HPM | 2019 | December | 0.9863 | 0.3785 |
| 1680 | HPM | 2019 | December | 0.2110 | 1.7623 |
| 1681 | HPM | 2019 | December | 0.9231 | 0.9710 |
| 1682 | HPM | 2019 | December | 0.9658 | 0.6881 |
| 1683 | HPM | 2019 | December | 0.8636 | 1.4349 |
| 1684 | HPM | 2019 | December | 0.8166 | 1.3732 |
| 1685 | HPM | 2019 | December | 0.9418 | 0.7997 |
| 1686 | HPM | 2019 | December | 0.8997 | 1.1006 |
| 1687 | HPM | 2019 | December | 0.9424 | 0.8161 |
| 1688 | HPM | 2019 | December | 0.9400 | 0.8498 |
| 1689 | HPM | 2019 | December | 0.9095 | 0.9644 |
| 1690 | HPM | 2019 | December | 0.9279 | 0.9444 |
| 1691 | HPM | 2019 | December | 0.9200 | 1.0540 |
| 1692 | HPM | 2019 | December | 0.8982 | 1.1124 |
| 1693 | HPM | 2019 | December | 0.7568 | 1.3589 |
| 1694 | HPM | 2019 | December | 0.9358 | 0.8940 |
| 1695 | HPM | 2019 | December | 0.9392 | 0.8820 |
| 1696 | HPM | 2019 | December | 0.9424 | 0.8277 |
| 1697 | HPM | 2019 | December | 0.9218 | 0.9593 |
| 1698 | HPM | 2019 | December | 0.8664 | 1.0459 |
| 1699 | HPM | 2019 | December | 0.9879 | 0.2880 |
| 1700 | HPM | 2019 | December | 0.8140 | 1.3247 |
| 1701 | HPM | 2019 | December | 0.8831 | 1.1228 |
| 1702 | HPM | 2019 | December | 0.9540 | 0.6280 |
| 1703 | HPM | 2019 | December | 0.1510 | 1.7942 |
| 1704 | HPM | 2019 | December | 0.2549 | 1.5940 |
| 1705 | HPM | 2019 | December | 0.7482 | 1.5464 |
| 1706 | HPM | 2019 | December | 0.9526 | 0.7294 |
| 1707 | HPM | 2019 | December | 0.9411 | 0.8633 |
| 1708 | HPM | 2019 | December | 0.8704 | 0.9824 |
| 1709 | HPM | 2019 | December | 0.8745 | 1.1448 |
| 1710 | HPM | 2019 | December | 0.9228 | 1.0295 |
| 1711 | HPM | 2019 | December | 0.5574 | 1.8869 |
| 1712 | HPM | 2019 | December | 0.6723 | 1.8097 |
| 1713 | HPM | 2019 | December | 0.8909 | 0.8873 |
| 1714 | HPM | 2019 | December | 0.9660 | 0.6225 |
| 1715 | HPM | 2019 | December | 0.8954 | 0.9771 |
| 1716 | HPM | 2019 | December | 0.9383 | 0.8265 |
| 1717 | HPM | 2019 | December | 0.9403 | 0.7435 |
| 1718 | HPM | 2019 | December | 0.8977 | 1.0758 |
| 1719 | HPM | 2019 | December | 0.8738 | 1.0945 |
| 1720 | HPM | 2019 | December | 0.8873 | 1.2603 |
| 1721 | HPM | 2019 | December | 0.9126 | 0.9924 |
| 1722 | HPM | 2019 | December | 0.8324 | 1.2109 |
| 1723 | HPM | 2019 | December | 0.7651 | 1.3259 |
| 1724 | HPM | 2019 | December | 0.9075 | 1.1668 |
| 1725 | HPM | 2019 | December | 0.9146 | 1.0067 |
| 1726 | HPM | 2019 | December | 0.9278 | 0.9742 |
| 1727 | HPM | 2019 | December | 0.9676 | 0.6833 |
| 1728 | HPM | 2019 | December | 0.9660 | 0.6090 |
| 1729 | HPM | 2019 | December | 0.8810 | 0.9463 |
| 1730 | HPM | 2019 | December | 0.9972 | 0.2048 |
| 1731 | HPM | 2019 | December | 0.9492 | 0.8097 |
| 1732 | HPM | 2019 | December | 0.9203 | 0.9793 |
| 1733 | HPM | 2019 | December | 0.9135 | 1.0333 |
| 1734 | HPM | 2019 | December | 0.9280 | 0.9165 |
| 1735 | HPM | 2019 | December | 0.9727 | 0.5602 |
| 1736 | HPM | 2019 | December | 0.9965 | 0.2076 |
| 1737 | HPM | 2019 | December | 0.9718 | 0.5793 |
| 1738 | HPM | 2019 | December | 0.9643 | 0.6397 |
| 1739 | HPM | 2019 | December | 0.6687 | 1.7415 |
| 1740 | HPM | 2019 | December | 0.8921 | 1.0341 |
| 1741 | HPM | 2019 | December | 0.6310 | 1.8408 |
| 1742 | HPM | 2019 | December | 0.8747 | 1.1114 |
| 1743 | HPM | 2019 | December | 0.5839 | 1.9733 |
| 1744 | PK | 2018 | December | 0.6398 | 1.8336 |
| 1745 | PK | 2018 | December | 0.7034 | 1.8252 |
| 1746 | PK | 2018 | December | 0.5524 | 1.8725 |
| 1747 | PK | 2018 | December | 0.5263 | 1.9354 |
| 1748 | PK | 2018 | December | 0.3446 | 1.9148 |
| 1749 | PK | 2018 | December | 0.7650 | 1.5394 |
| 1750 | PK | 2018 | December | 0.7022 | 1.7208 |
| 1751 | PK | 2018 | December | 0.8269 | 1.3812 |
| 1752 | PK | 2018 | December | 0.7174 | 1.7474 |
| 1753 | PK | 2018 | December | 0.9064 | 1.0217 |
| 1754 | PK | 2018 | December | 0.2194 | 1.6609 |
| 1755 | PK | 2018 | December | 0.7910 | 1.4333 |
| 1756 | PK | 2018 | December | 0.1524 | 2.1742 |
| 1757 | PK | 2018 | December | 0.3605 | 1.9971 |
| 1758 | PK | 2018 | December | 0.4025 | 2.4006 |
| 1759 | PK | 2018 | December | 0.4214 | 2.1868 |
| 1760 | PK | 2018 | December | 0.6108 | 1.8930 |
| 1761 | PK | 2018 | December | 0.3076 | 2.1641 |
| 1762 | PK | 2018 | December | 0.4471 | 2.2740 |
| 1763 | PK | 2018 | December | 0.5838 | 2.1618 |
| 1764 | PK | 2018 | December | 0.6229 | 1.6082 |
| 1765 | PK | 2018 | December | 0.4383 | 2.3889 |
| 1766 | PK | 2018 | December | 0.6328 | 2.0140 |
| 1767 | PK | 2018 | December | 0.6454 | 1.9595 |
| 1768 | PK | 2018 | December | 0.9009 | 1.1563 |
| 1769 | PK | 2018 | December | 0.9686 | 0.6010 |
| 1770 | PK | 2018 | December | 0.6754 | 1.8555 |
| 1771 | PK | 2018 | December | 0.4545 | 2.3832 |
| 1772 | PK | 2018 | December | 0.4274 | 1.9810 |
| 1773 | PK | 2018 | December | 0.2313 | 2.5891 |
| 1774 | PK | 2018 | December | 0.8605 | 1.1774 |
| 1775 | PK | 2018 | December | 0.7875 | 1.5736 |
| 1776 | PK | 2018 | December | 0.1902 | 1.9046 |
| 1777 | PK | 2018 | December | 0.1589 | 2.1927 |
| 1778 | PK | 2018 | December | 0.4731 | 2.2837 |
| 1779 | PK | 2018 | December | 0.0688 | 1.5279 |
| 1780 | PK | 2018 | December | 0.7925 | 1.5771 |
| 1781 | PK | 2018 | December | 0.5975 | 1.7736 |
| 1782 | PK | 2018 | December | 0.3063 | 2.0823 |
| 1783 | PK | 2018 | December | 0.7234 | 1.6769 |
| 1784 | PK | 2018 | December | 0.6108 | 2.0678 |
| 1785 | PK | 2018 | December | 0.3731 | 2.3020 |
| 1786 | PK | 2018 | December | 0.3821 | 1.7168 |
| 1787 | PK | 2018 | December | 0.1780 | 1.4881 |
| 1788 | PK | 2018 | December | 0.7968 | 1.6361 |
| 1789 | PK | 2018 | December | 0.0416 | 1.2651 |
| 1790 | PK | 2018 | December | 0.2626 | 1.6777 |
| 1791 | PK | 2018 | December | 0.5437 | 1.9464 |
| 1792 | PK | 2018 | December | 0.1799 | 2.0484 |
| 1793 | PK | 2018 | December | 0.2973 | 1.9666 |
| 1794 | PK | 2018 | December | 0.2353 | 1.5273 |
| 1795 | PK | 2018 | December | 0.8046 | 1.4701 |
| 1796 | PK | 2018 | December | 0.6835 | 2.0942 |
| 1797 | PK | 2018 | December | 0.1443 | 1.6460 |
| 1798 | PK | 2018 | December | 0.0820 | 1.2911 |
| 1799 | PK | 2018 | December | 0.9842 | 0.4131 |
| 1800 | PK | 2018 | December | 0.9262 | 0.9892 |
| 1801 | PK | 2018 | December | 0.8242 | 1.4959 |
| 1802 | PK | 2018 | December | 0.8231 | 1.6144 |
| 1803 | PK | 2018 | December | 0.0768 | 1.7714 |
| 1804 | PK | 2018 | December | 0.6379 | 1.7907 |
| 1805 | PK | 2018 | December | 0.6300 | 1.9385 |
| 1806 | PK | 2018 | December | 0.8978 | 1.1027 |
| 1807 | PK | 2018 | December | 0.3794 | 2.0174 |
| 1808 | PK | 2018 | December | 0.5138 | 2.0342 |
| 1809 | PK | 2018 | December | 0.3093 | 2.2640 |
| 1810 | PK | 2018 | December | 0.3889 | 2.1629 |
| 1811 | PK | 2018 | December | 0.6064 | 1.0186 |
| 1812 | PK | 2018 | December | 0.8266 | 0.7845 |
| 1813 | PK | 2018 | December | 0.9823 | 0.4449 |
| 1814 | PK | 2018 | December | 0.3722 | 1.9596 |
| 1815 | PK | 2018 | December | 0.3927 | 2.2224 |
| 1816 | PK | 2018 | December | 0.4674 | 2.3871 |
| 1817 | PK | 2018 | December | 0.5449 | 2.3425 |
| 1818 | PK | 2018 | December | 0.1804 | 2.1367 |
| 1819 | PK | 2018 | December | 0.3496 | 1.4675 |
| 1820 | PK | 2018 | December | 0.2352 | 1.6889 |
| 1821 | PK | 2018 | December | 0.8012 | 1.6499 |
| 1822 | PK | 2018 | December | 0.6871 | 1.7478 |
| 1823 | PK | 2018 | December | 0.3975 | 1.9239 |
| 1824 | PK | 2018 | December | 0.5463 | 2.2492 |
| 1825 | PK | 2018 | December | 0.2536 | 1.8405 |
| 1826 | PK | 2018 | December | 0.5348 | 1.7388 |
| 1827 | PK | 2018 | December | 0.6380 | 1.8136 |
| 1828 | PK | 2018 | December | 0.6244 | 1.7877 |
| 1829 | PK | 2018 | December | 0.4424 | 1.9009 |
| 1830 | PK | 2018 | December | 0.1670 | 1.5913 |
| 1831 | PK | 2018 | December | 0.4593 | 2.1601 |
| 1832 | PK | 2018 | December | 0.2440 | 2.0513 |
| 1833 | PK | 2018 | December | 0.6196 | 2.0437 |
| 1834 | PK | 2018 | December | 0.6382 | 1.9044 |
| 1835 | PK | 2018 | December | 0.1822 | 1.6184 |
| 1836 | PK | 2018 | December | 0.4920 | 2.1183 |
| 1837 | PK | 2018 | December | 0.5565 | 2.1519 |
| 1838 | PK | 2018 | December | 0.2847 | 2.0649 |
| 1839 | PK | 2018 | December | 0.5821 | 2.1817 |
| 1840 | PK | 2018 | December | 0.3821 | 2.1012 |
| 1841 | PK | 2018 | December | 0.5954 | 1.7349 |
| 1842 | PK | 2018 | December | 0.4467 | 2.3639 |
| 1843 | PK | 2018 | December | 0.0535 | 1.1949 |
| 1844 | PK | 2018 | December | 0.8866 | 1.0407 |
| 1845 | PK | 2018 | December | 0.3508 | 1.6240 |
| 1846 | PK | 2018 | December | 0.7779 | 1.2155 |
| 1847 | PK | 2018 | December | 0.6352 | 1.6852 |
| 1848 | PK | 2018 | December | 0.6015 | 2.1666 |
| 1849 | PK | 2018 | December | 0.6352 | 2.1354 |
| 1850 | PK | 2018 | December | 0.5739 | 1.9873 |
| 1851 | PK | 2018 | December | 0.9067 | 1.0709 |
| 1852 | PK | 2018 | December | 0.9983 | 0.1692 |
| 1853 | PK | 2018 | December | 0.9798 | 0.5849 |
| 1854 | PK | 2018 | December | 0.5721 | 2.0876 |
| 1855 | PK | 2018 | December | 0.4120 | 2.0865 |
| 1856 | PK | 2018 | December | 0.2537 | 1.6970 |
| 1857 | PK | 2018 | December | 0.4184 | 2.2660 |
| 1858 | PK | 2018 | December | 0.5043 | 2.1461 |
| 1859 | PK | 2018 | December | 0.0996 | 1.6142 |
| 1860 | PK | 2018 | December | 0.4694 | 2.2368 |
| 1861 | PK | 2018 | December | 0.0653 | 1.3163 |
| 1862 | PK | 2018 | December | 0.5994 | 1.9659 |
| 1863 | PK | 2018 | December | 0.6965 | 1.8263 |
| 1864 | PK | 2018 | December | 0.6690 | 1.9725 |
| 1865 | PK | 2018 | December | 0.6658 | 1.9064 |
| 1866 | PK | 2018 | December | 0.3583 | 2.1704 |
| 1867 | PK | 2018 | December | 0.3637 | 2.1659 |
| 1868 | PK | 2018 | December | 0.0599 | 1.4241 |
| 1869 | PK | 2018 | December | 0.0547 | 1.3641 |
| 1870 | PK | 2018 | December | 0.2156 | 1.5968 |
| 1871 | PK | 2018 | December | 0.3334 | 2.4105 |
| 1872 | PK | 2018 | December | 0.2296 | 1.1663 |
| 1873 | PK | 2018 | December | 0.3020 | 1.1092 |
| 1874 | PK | 2019 | April | 0.3572 | 2.1451 |
| 1875 | PK | 2019 | April | 0.4225 | 1.7427 |
| 1876 | PK | 2019 | April | 0.1948 | 1.6897 |
| 1877 | PK | 2019 | April | 0.0712 | 1.6208 |
| 1878 | PK | 2019 | April | 0.2217 | 1.6207 |
| 1879 | PK | 2019 | April | 0.1408 | 2.3141 |
| 1880 | PK | 2019 | April | 0.0197 | 1.9541 |
| 1881 | PK | 2019 | April | 0.1195 | 2.1511 |
| 1882 | PK | 2019 | April | 0.4751 | 1.7121 |
| 1883 | PK | 2019 | April | 0.0771 | 2.3698 |
| 1884 | PK | 2019 | April | 0.1223 | 1.8783 |
| 1885 | PK | 2019 | April | 0.1289 | 2.2116 |
| 1886 | PK | 2019 | April | 0.1032 | 1.8486 |
| 1887 | PK | 2019 | April | 0.4275 | 2.1314 |
| 1888 | PK | 2019 | April | 0.2675 | 1.9685 |
| 1889 | PK | 2019 | April | 0.2132 | 2.0729 |
| 1890 | PK | 2019 | April | 0.5256 | 2.0791 |
| 1891 | PK | 2019 | April | 0.7405 | 1.4601 |
| 1892 | PK | 2019 | April | 0.5867 | 1.9112 |
| 1893 | PK | 2019 | April | 0.6758 | 1.5874 |
| 1894 | PK | 2019 | April | 0.9984 | 0.0889 |
| 1895 | PK | 2019 | April | 0.6326 | 1.3789 |
| 1896 | PK | 2019 | April | 0.8434 | 0.8847 |
| 1897 | PK | 2019 | April | 0.9726 | 0.3930 |
| 1898 | PK | 2019 | April | 0.6468 | 1.1370 |
| 1899 | PK | 2019 | April | 0.4098 | 1.3525 |
| 1900 | PK | 2019 | April | 0.8215 | 0.9221 |
| 1901 | PK | 2019 | April | 0.9980 | 0.1003 |
| 1902 | PK | 2019 | April | 0.5385 | 1.3157 |
| 1903 | PK | 2019 | April | 0.7803 | 1.1780 |
| 1904 | PK | 2019 | April | 0.7218 | 1.2888 |
| 1905 | PK | 2019 | April | 0.3837 | 1.4842 |
| 1906 | PK | 2019 | April | 0.9993 | 0.0941 |
| 1907 | PK | 2019 | April | 0.7018 | 1.0428 |
| 1908 | PK | 2019 | April | 0.9040 | 0.7898 |
| 1909 | PK | 2019 | April | 0.8989 | 1.1830 |
| 1910 | PK | 2019 | April | 0.8401 | 0.9183 |
| 1911 | PK | 2019 | April | 0.7988 | 0.9754 |
| 1912 | PK | 2019 | April | 0.9669 | 0.6202 |
| 1913 | PK | 2019 | April | 0.9770 | 0.3494 |
| 1914 | PK | 2019 | April | 0.7842 | 1.2919 |
| 1915 | PK | 2019 | April | 0.6324 | 1.4194 |
| 1916 | PK | 2019 | April | 0.7724 | 1.1678 |
| 1917 | PK | 2019 | April | 0.0961 | 1.1604 |
| 1918 | PK | 2019 | April | 0.3017 | 1.4676 |
| 1919 | PK | 2019 | April | 0.9676 | 0.5400 |
| 1920 | PK | 2019 | April | 0.8687 | 1.0782 |
| 1921 | PK | 2019 | April | 0.8691 | 1.2541 |
| 1922 | PK | 2019 | April | 0.6651 | 1.1404 |
| 1923 | PK | 2019 | April | 0.7836 | 1.3883 |
| 1924 | PK | 2019 | April | 0.6159 | 1.5164 |
| 1925 | PK | 2019 | April | 0.6795 | 1.3451 |
| 1926 | PK | 2019 | April | 0.2291 | 1.4552 |
| 1927 | PK | 2019 | April | 0.4916 | 1.3334 |
| 1928 | PK | 2019 | April | 0.6846 | 1.1902 |
| 1929 | PK | 2019 | April | 0.3523 | 1.4413 |
| 1930 | PK | 2019 | April | 0.6789 | 1.2025 |
| 1931 | PK | 2019 | April | 0.7899 | 1.1206 |
| 1932 | PK | 2019 | April | 0.7423 | 1.2854 |
| 1933 | PK | 2019 | April | 0.3694 | 1.2934 |
| 1934 | PK | 2019 | April | 0.9984 | 0.1334 |
| 1935 | PK | 2019 | April | 0.9989 | 0.0967 |
| 1936 | PK | 2019 | April | 0.9960 | 0.1754 |
| 1937 | PK | 2019 | April | 0.7990 | 0.9593 |
| 1938 | PK | 2019 | April | 0.6175 | 1.3396 |
| 1939 | PK | 2019 | April | 0.6970 | 1.2612 |
| 1940 | PK | 2019 | April | 0.5634 | 1.7318 |
| 1941 | PK | 2019 | April | 0.6872 | 1.2592 |
| 1942 | PK | 2019 | April | 0.8119 | 1.0598 |
| 1943 | PK | 2019 | April | 0.9903 | 0.2462 |
| 1944 | PK | 2019 | April | 0.4494 | 1.3246 |
| 1945 | PK | 2019 | April | 0.9519 | 0.6246 |
| 1946 | PK | 2019 | April | 0.8230 | 1.3785 |
| 1947 | PK | 2019 | April | 0.7879 | 1.2880 |
| 1948 | PK | 2019 | April | 0.3293 | 1.4142 |
| 1949 | PK | 2019 | April | 0.1666 | 1.4159 |
| 1950 | PK | 2019 | April | 0.8557 | 1.1975 |
| 1951 | PK | 2019 | April | 0.6476 | 1.2288 |
| 1952 | PK | 2019 | April | 0.8009 | 1.1160 |
| 1953 | PK | 2019 | April | 0.8727 | 0.9794 |
| 1954 | PK | 2019 | April | 0.6874 | 1.2361 |
| 1955 | PK | 2019 | April | 0.7712 | 0.9853 |
| 1956 | PK | 2019 | April | 0.7654 | 1.1462 |
| 1957 | PK | 2019 | April | 0.7595 | 1.1864 |
| 1958 | PK | 2019 | April | 0.8033 | 1.3348 |
| 1959 | PK | 2019 | April | 0.4860 | 1.3239 |
| 1960 | PK | 2019 | April | 0.8188 | 1.2594 |
| 1961 | PK | 2019 | April | 0.8328 | 1.3552 |
| 1962 | PK | 2019 | April | 0.7853 | 1.2951 |
| 1963 | PK | 2019 | April | 0.6315 | 1.2915 |
| 1964 | PK | 2019 | April | 0.3414 | 1.3163 |
| 1965 | PK | 2019 | April | 0.7106 | 1.1124 |
| 1966 | PK | 2019 | April | 0.8466 | 1.1373 |
| 1967 | PK | 2019 | April | 0.7542 | 1.2266 |
| 1968 | PK | 2019 | April | 0.7510 | 1.2140 |
| 1969 | PK | 2019 | April | 0.7556 | 0.9994 |
| 1970 | PK | 2019 | April | 0.7911 | 1.1011 |
| 1971 | PK | 2019 | April | 0.5379 | 1.3130 |
| 1972 | PK | 2019 | April | 0.3386 | 1.4011 |
| 1973 | PK | 2019 | April | 0.8790 | 0.8836 |
| 1974 | PK | 2019 | April | 0.5285 | 1.4298 |
| 1975 | PK | 2019 | April | 0.5189 | 1.2804 |
| 1976 | PK | 2019 | April | 0.9091 | 0.9389 |
| 1977 | PK | 2019 | April | 0.2562 | 1.3552 |
| 1978 | PK | 2019 | April | 0.8582 | 1.0761 |
| 1979 | PK | 2019 | April | 0.7523 | 1.2990 |
| 1980 | PK | 2019 | April | 0.5496 | 1.4178 |
| 1981 | PK | 2019 | April | 0.2829 | 1.3326 |
| 1982 | PK | 2019 | April | 0.8837 | 0.9157 |
| 1983 | PK | 2019 | April | 0.2741 | 1.2812 |
| 1984 | PK | 2019 | April | 0.7760 | 1.1530 |
| 1985 | PK | 2019 | April | 0.1400 | 1.1671 |
| 1986 | PK | 2019 | April | 0.7098 | 1.1626 |
| 1987 | PK | 2019 | April | 0.4545 | 1.5224 |
| 1988 | PK | 2019 | April | 0.8459 | 1.0678 |
| 1989 | PK | 2019 | April | 0.6638 | 1.4473 |
| 1990 | PK | 2019 | April | 0.2317 | 1.4359 |
| 1991 | PK | 2019 | April | 0.6864 | 0.9891 |
| 1992 | PK | 2019 | April | 0.7444 | 1.0540 |
| 1993 | PK | 2019 | April | 0.8073 | 1.0925 |
| 1994 | PK | 2019 | April | 0.7734 | 0.9859 |
| 1995 | PK | 2019 | April | 0.7154 | 1.1260 |
| 1996 | PK | 2019 | April | 0.8587 | 0.9173 |
| 1997 | PK | 2019 | April | 0.3860 | 1.1212 |
| 1998 | PK | 2019 | April | 0.1086 | 1.1323 |
| 1999 | PK | 2019 | April | 0.8429 | 0.9605 |
| 2000 | PK | 2019 | April | 0.7525 | 1.1065 |
| 2001 | PK | 2019 | April | 0.7702 | 1.0708 |
| 2002 | PK | 2019 | April | 0.6681 | 1.0565 |
| 2003 | PK | 2019 | April | 0.9194 | 0.9401 |
| 2004 | PK | 2019 | December | 0.5258 | 2.0209 |
| 2005 | PK | 2019 | December | 0.4827 | 1.6348 |
| 2006 | PK | 2019 | December | 0.6933 | 1.5944 |
| 2007 | PK | 2019 | December | 0.6949 | 1.5490 |
| 2008 | PK | 2019 | December | 0.7549 | 1.4920 |
| 2009 | PK | 2019 | December | 0.9830 | 0.4363 |
| 2010 | PK | 2019 | December | 0.6434 | 1.4821 |
| 2011 | PK | 2019 | December | 0.5468 | 1.5963 |
| 2012 | PK | 2019 | December | 0.8092 | 1.1326 |
| 2013 | PK | 2019 | December | 0.9812 | 0.4688 |
| 2014 | PK | 2019 | December | 0.9196 | 0.9485 |
| 2015 | PK | 2019 | December | 0.9441 | 0.8105 |
| 2016 | PK | 2019 | December | 0.8007 | 1.5188 |
| 2017 | PK | 2019 | December | 0.4282 | 1.7733 |
| 2018 | PK | 2019 | December | 0.8319 | 1.3333 |
| 2019 | PK | 2019 | December | 0.6153 | 2.1177 |
| 2020 | PK | 2019 | December | 0.6479 | 1.9540 |
| 2021 | PK | 2019 | December | 0.7363 | 1.7678 |
| 2022 | PK | 2019 | December | 0.6068 | 2.0607 |
| 2023 | PK | 2019 | December | 0.6040 | 2.1146 |
| 2024 | PK | 2019 | December | 0.8366 | 1.4676 |
| 2025 | PK | 2019 | December | 0.9062 | 1.1531 |
| 2026 | PK | 2019 | December | 0.9906 | 0.3425 |
| 2027 | PK | 2019 | December | 0.9501 | 0.8200 |
| 2028 | PK | 2019 | December | 0.9974 | 0.1968 |
| 2029 | PK | 2019 | December | 0.9892 | 0.4062 |
| 2030 | PK | 2019 | December | 0.9176 | 1.1251 |
| 2031 | PK | 2019 | December | 0.4691 | 2.2649 |
| 2032 | PK | 2019 | December | 0.9437 | 0.8959 |
| 2033 | PK | 2019 | December | 0.6771 | 2.0157 |
| 2034 | PK | 2019 | December | 0.4026 | 2.3150 |
| 2035 | PK | 2019 | December | 0.3726 | 2.3778 |
| 2036 | PK | 2019 | December | 0.7219 | 1.9670 |
| 2037 | PK | 2019 | December | 0.7580 | 1.8858 |
| 2038 | PK | 2019 | December | 0.3728 | 2.1803 |
| 2039 | PK | 2019 | December | 0.3063 | 1.9433 |
| 2040 | PK | 2019 | December | 0.6277 | 1.9339 |
| 2041 | PK | 2019 | December | 0.7788 | 1.7889 |
| 2042 | PK | 2019 | December | 0.9070 | 1.2250 |
| 2043 | PK | 2019 | December | 0.8835 | 1.3701 |
| 2044 | PK | 2019 | December | 0.1144 | 1.8536 |
| 2045 | PK | 2019 | December | 0.3333 | 2.2265 |
| 2046 | PK | 2019 | December | 0.6319 | 2.2004 |
| 2047 | PK | 2019 | December | 0.8274 | 1.4832 |
| 2048 | PK | 2019 | December | 0.4193 | 1.9874 |
| 2049 | PK | 2019 | December | 0.2197 | 2.1690 |
| 2050 | PK | 2019 | December | 0.5289 | 2.0453 |
| 2051 | PK | 2019 | December | 0.2333 | 1.9981 |
| 2052 | PK | 2019 | December | 0.2442 | 2.3584 |
| 2053 | PK | 2019 | December | 0.6349 | 2.1970 |
| 2054 | PK | 2019 | December | 0.7291 | 1.6639 |
| 2055 | PK | 2019 | December | 0.6799 | 2.0546 |
| 2056 | PK | 2019 | December | 0.5700 | 2.0077 |
| 2057 | PK | 2019 | December | 0.1885 | 1.9151 |
| 2058 | PK | 2019 | December | 0.3261 | 1.4981 |
| 2059 | PK | 2019 | December | 0.3943 | 2.3303 |
| 2060 | PK | 2019 | December | 0.5321 | 2.2305 |
| 2061 | PK | 2019 | December | 0.5032 | 1.8285 |
| 2062 | PK | 2019 | December | 0.5490 | 2.1896 |
| 2063 | PK | 2019 | December | 0.5970 | 2.0670 |
| 2064 | PK | 2019 | December | 0.3426 | 1.4317 |
| 2065 | PK | 2019 | December | 0.9833 | 0.5230 |
| 2066 | PK | 2019 | December | 0.7599 | 1.7469 |
| 2067 | PK | 2019 | December | 0.9281 | 1.0005 |
| 2068 | PK | 2019 | December | 0.7238 | 1.5704 |
| 2069 | PK | 2019 | December | 0.4179 | 1.6344 |
| 2070 | PK | 2019 | December | 0.2806 | 2.2180 |
| 2071 | PK | 2019 | December | 0.6287 | 1.6629 |
| 2072 | PK | 2019 | December | 0.7406 | 1.7840 |
| 2073 | PK | 2019 | December | 0.7901 | 1.5933 |
| 2074 | PK | 2019 | December | 0.2299 | 1.8337 |
| 2075 | PK | 2019 | December | 0.9355 | 0.9353 |
| 2076 | PK | 2019 | December | 0.5999 | 2.1226 |
| 2077 | PK | 2019 | December | 0.5056 | 2.3793 |
| 2078 | PK | 2019 | December | 0.4137 | 2.4332 |
| 2079 | PK | 2019 | December | 0.6013 | 2.1085 |
| 2080 | PK | 2019 | December | 0.4194 | 2.3278 |
| 2081 | PK | 2019 | December | 0.0931 | 1.9045 |
| 2082 | PK | 2019 | December | 0.1230 | 1.7529 |
| 2083 | PK | 2019 | December | 0.5502 | 2.2488 |
| 2084 | PK | 2019 | December | 0.5575 | 2.3230 |
| 2085 | PK | 2019 | December | 0.6923 | 1.9471 |
| 2086 | PK | 2019 | December | 0.2368 | 2.0096 |
| 2087 | PK | 2019 | December | 0.4196 | 2.0472 |
| 2088 | PK | 2019 | December | 0.2973 | 2.0432 |
| 2089 | PK | 2019 | December | 0.5753 | 2.2849 |
| 2090 | PK | 2019 | December | 0.7137 | 2.0447 |
| 2091 | PK | 2019 | December | 0.4269 | 2.1271 |
| 2092 | PK | 2019 | December | 0.5605 | 2.2347 |
| 2093 | PK | 2019 | December | 0.3383 | 2.1466 |
| 2094 | PK | 2019 | December | 0.8781 | 1.2197 |
| 2095 | PK | 2019 | December | 0.9810 | 0.5276 |
| 2096 | PK | 2019 | December | 0.2922 | 2.1218 |
| 2097 | PK | 2019 | December | 0.6645 | 1.7902 |
| 2098 | PK | 2019 | December | 0.4318 | 2.2372 |
| 2099 | PK | 2019 | December | 0.2583 | 2.0029 |
| 2100 | PK | 2019 | December | 0.9776 | 0.4701 |
| 2101 | PK | 2019 | December | 0.9208 | 1.0532 |
| 2102 | PK | 2019 | December | 0.4119 | 2.2424 |
| 2103 | PK | 2019 | December | 0.3291 | 1.7196 |
